# FOXD1, a hypoxia-related gene, accelerates prostate cancer cell growth by increasing glycolysis under hypoxia conditions

**DOI:** 10.1186/s12896-025-01061-6

**Published:** 2025-11-10

**Authors:** Jing Gao, Shuang Wu

**Affiliations:** 1 Department of Urology, The First Affiliated Hospital of Jinzhou Medical University, Jinzhou, China; 2 Department of Blood Transfusion, The First Affiliated Hospital of Jinzhou Medical University, Jinzhou, China

**Keywords:** Prostate cancer, Tumor microenvironment, Hypoxia, FOXD1, Glycolysis

## Abstract

Hypoxia is a key feature of the tumor microenvironment, and can promote tumor progression. FOXD1 has been found to be involved in several types of cancer; however, its role in mediating cancer cell growth under hypoxia conditions is unclear. In this study, we aimed to test the effect of FOXD1 on prostate cancer cell growth in response to hypoxia. Our results showed that FOXD1 expression was upregulated in prostate cancer. Under normoxia conditions, FOXD1 knockdown inhibited prostate cancer cell viability and colony formation, while FOXD1 overexpression exhibited the opposite effects. Hypoxia exposure increased the expression of FOXD1 in prostate cancer. FOXD1 knockdown inhibited hypoxia-induced increase in cell viability and colony formation ability, whereas FOXD1 overexpression showed the opposite effects. In addition, FOXD1 knockdown inhibited hypoxia-induced increase in lactate production, glucose consumption, and the expression levels of HK-2 and LDHA. Moreover, inhibition of glycolysis by 2-deoxyglucose reversed the effect of FOXD1 overexpression on hypoxia-induced prostate cancer cell growth. These results indicated that hypoxia-induced FOXD1 expression accelerated glycolysis, ultimately promoting prostate cancer cell growth.

**Supplementary Information:**

The online version contains supplementary material available at 10.1186/s12896-025-01061-6.

## Introduction

Prostate cancer is one of the most common malignancies among middle-aged men with increased mortality rates in all racial and ethnic groups [1]. Currently, treatment for prostate cancer includes active surveillance, androgen deprivation therapy, radical prostatectomy, and ablative radiotherapy [2]. Available combination therapy is now effective for localized prostate cancer; however, like any solid tumor, intratumoral hypoxia, as a common feature of prostate cancer, often leads to treatment resistance and poor prognosis [3–5]. Improved management for prostate cancer is still needed currently.

In general, hypoxia is a typical tumor microenvironment feature nearly all solid tumors, which is closely associated with immune evasion, angiogenesis, cancer stem cell specification, cancer cell motility, extracellular matrix remodeling, and metabolic reprogramming [6–9]. Increasing evidence has shown that hypoxia exerts critical roles in maintaining prostate cancer progression [10, 11]. Therefore, targeting hypoxia might be a promising strategy for improving the clinical outcomes of prostate cancer patients and enhancing the efficacy of cancer immunotherapy.

Forkhead box D1 (FOXD1), a new member of the FOX transcription factor family, has been found to serve as a key mediator in several biological processes such as cell reprogramming, embryo implantation, and kidney and retina development [12]. Numerous studies have shown that FOXD1 dysregulation is linked to the development of diverse diseases, including recurrent pregnancy loss [13], renal fibrosis [14], liver fibrosis [15], and cancer [16]. Particularly, FOXD1 acts as an oncogene in mediating the onset and progression of numerous human cancers [16]. Currently, FOXD1 has been found to be involved in the tumorigenesis of prostate cancer. Huang et al. reported that FOXD1 expression is upregulated in prostate cancer tissues and its high expression is associated with clinical stage and survival in patients with prostate cancer [17]. Another study suggests that silencing of FOXD1 suppresses proliferation, migration, and invasion of the prostate cancer [18]. These findings suggest that FOXD1 might be a prognostic biomarker and therapeutic target for prostate cancer. However, the role of FOXD1 in regulating cancer development under hypoxia conditions has not been reported.

In the current study, we aimed to test the effect of FOXD1 on prostate cancer cell growth in response to hypoxia. We determined whether FOXD1 is a hypoxia-related gene, and explored the roles of FOXD1 in prostate cancer cell growth and glycolysis.

## Methods and materials

### Identification of differentially expressed genes (DEGs) related to hypoxia in prostate cancer

The GSE259218, GSE200204, GSE80657, GSE53012, and GSE78245 datasets from the GEO database (https://www.ncbi.nlm.nih.gov/geo/) were used to analyze DEGs related to hypoxia in different prostate cancer cell lines.

### Acquisition of DEGs in patients with prostate cancer

The mRNA expression of BNIP3L, C4orf3, P4HA2, FOXD1, RAB31, and HK2 was obtained from GEPIA online tool which analyzes the mRNA expression data tumors and normal samples from The Cancer Genome Atlas (TCGA) databases and the Genotype-Tissue Expression (GTEx) projects [19]. The GSE6919-GPL8300, GSE88808, GSE32571, GSE46602, GSE55945, and GSE70768 datasets from the GEO database were used to assess the change of FOXD1 expression in prostate cancer and control samples.

### Cell culture and treatments

The human prostate epithelial cell line RWPE-1 (CRL-11609; ATCC, Manassas, VA, USA) was maintained in K-SFM medium (Gibco, Grand Island, NY, USA). Two human prostate cancer cell lines PC-3 (CRL-1435; ATCC) and 22Rv1 (CRL-2505; ATCC) were maintained in F-12 K (30-2004; ATCC) and RPMI-1640 medium (30-2001; ATCC), respectively. All mediums contain 10% fetal bovine serum (Hyclone, Logan, UT, USA) and 1% penicillin/streptomycin (Hyclone). All cell lines were cultured in a humidified atmosphere with 5% CO_2_ at 37 °C. To create hypoxic conditions, cells were cultured under hypoxia conditions (1% O_2_, 5% CO_2_, and 94% N_2_) [20, 21]. Cells in normoxia control group were incubated with 5% CO_2_ and ambient O_2_. To inhibit glycolysis, cells were treated with 5 mM 2-deoxyglucose (2-DG; Sigma-Aldrich, St. Louis, MO, USA) [22].

Short hairpin RNA (shRNA) targeting FOXD1 (sh-FOXD1-1 and sh-FOXD1-2), non-targeting shRNA (sh-con), pcDNA3.1 empty vector (pcDNA), and FOXD1 overexpression plasmid vector (pcDNA-FOXD1) were purchased from RiboBio (Guangzhou, China). PC-3 and 22Rv1 cells were transfected with sh-con, sh-FOXD1-1, sh-FOXD1-2, pcDNA, or pcDNA-FOXD1 using Lipofectamine™ 2000 transfection reagent (Gibco Laboratories). After 72 h, the expression levels of FOXD1 were determined using western blotting.

### Quantitative real-time PCR (qRT-PCR)

Total RNAs were isolated from PC-3 and 22Rv1 cells using TRIzol reagent (Invitrogen, Carlsbad, CA, USA) and converted into cDNA with a Reverse Transcription Kit (Invitrogen). qPCR was performed with SYBR Green reagent (Bio-Rad, Hercules, CA, USA) on a Real-Time PCR Detection System (Bio-Rad). Relative gene expression was normalized to β-actin and calculated using the 2^−ΔΔCt^ method. The primers used for each gene are as follows: FOXD1, forward 5’-TGAGCACTGAGATGTCCGATG-3’ and reverse 5’-CACCACGTCGATGTCTGTTTC-3’; HK2, forward 5’-TGCCACCAGACTAAACTAGACG-3’ and reverse 5’-CCCGTGCCCACAATGAGAC-3’; LDHA, forward 5’-TTGACCTACGTGGCTTGGAAG-3’ and reverse 5’-GGTAACGGAATCGGGCTGAAT-3’; β-actin forward 5’-CATGTACGTTGCTATCCAGGC-3’ and reverse 5’-CTCCTTAATGTCACGCACGAT-3’. The experiments were independently repeated three times.

### Western blotting

Total protein samples of PC-3 and 22Rv1 cells were extracted with cell lysis buffer (Beyotime Biotechnology, Shanghai, China) containing protease and phosphatase inhibitors (Roche Diagnostics, Mannheim, Germany). After determination of the protein concentrations with a BCA Assay Kit (Beyotime Biotechnology), the samples were separated by 10% SDS-PAGE and subsequently transferred to PVDF membranes. Then the membranes were blocked with 5% BSA (Sigma-Aldrich) for 1 h, followed by incubation with primary antibody against FOXD1 (1:1000 dilution, PA5-35145; Invitrogen), hypoxia inducible factor 1 subunit alpha (HIF-1α; 1:1000 dilution, ab51608; Abcam, Cambridge, MA, USA), hexokinase-2 (HK-2; 1:1000 dilution, ab104836; Abcam), lactate dehydrogenase A (LDHA; 1:1000 dilution, #2012; CST, Danvers, MA, USA), and β-actin (1:2000 dilution, ab8227; Abcam) overnight at 4 °C. Finally, the membranes were exposed to secondary antibody dilution (1:3000 dilution, ab6721; Abcam) for 1 h at room temperature. The immune complexes were visualized by an enhanced chemiluminescence system (Thermo Fisher Scientific, Waltham, MA, USA). The quantitative analysis for each protein band was performed by ImageJ software (NIH, Bethesda, MD, USA). The experiments were independently repeated three times.

### Cell counting kit-8 (CCK-8) assay

PC-3 and 22Rv1 cells (3000 cells/well) were seeded into 96-well culture plates. After incubation for 72 h, 10 µL CCK-8 solution (Beyotime Biotechnology) was added to each well and then incubated for an additional 2 h. Lastly, the absorbance were read using a microplate reader (Bio-Tek Instruments, Winooski, VT, USA) with a 450 nm filter. The experiments were independently repeated three times and performed in triplicate.

### Colony formation assay

PC-3 and 22Rv1 cells were seeded in 6-well plates, followed by a culture for 10 days. Subsequently, the cells were fixed with 4% paraformaldehyde and then stained using 0.5% crystal violet for 15 min. The colonies > 100 μm were selected, photographed, and counted by Image J software (NIH). The experiments were independently repeated three times.

### Lactate production and glucose consumption measurement

Cells (2 × 10^5^ per well) were seeded onto 6-well plates and then subjected to different treatments. At the end of the experiments, lactate production and glucose uptake were evaluated by detecting the concentrations of lactate and glucose in cell culture supernatants with a Lactate Assay Kit (Nanjing Jiancheng Bioengineering, Nanjing, China) and Glucose Assay Kit (Rongsheng Biotech, Shanghai, China), according to the manufacturer’s protocol. The experiments were performed in triplicate and independently repeated three times.

### Statistical analysis

All experimental data in this study were analyzed using GraphPad Prism (version 8.0.2; GraphPad Software, San Diego, CA, USA). Data are expressed as the means ± SD or SEM. The normality of data distribution was evaluated using the Shapiro-Wilk test. For comparisons between two groups, the data were analyzed using Student’s t, Mann-Whitney U, or Wilcoxon signed-rank test. For comparisons between multiple groups, the data were analyzed using one-way ANOVA. A *p* value < 0.05 was considered as statistical significance.

## Results

### FOXD1 is identified as a hypoxia-related gene in prostate cancer

**Fig. 1:**
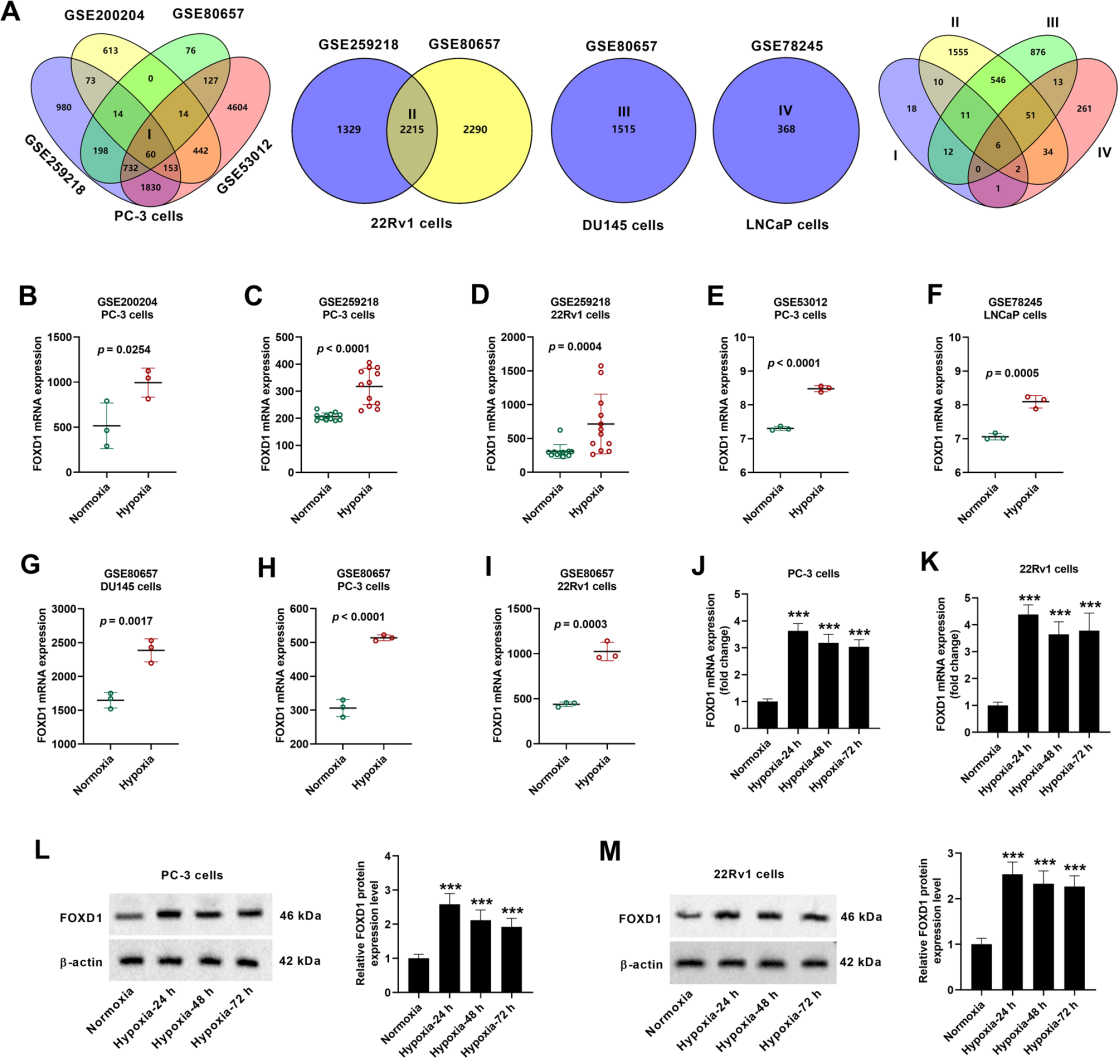
FOXD1 expression is upregulated in prostate cancer in response to hypoxia. (**A-I**) Analysis of hypoxia-related genes and FOXD1 mRNA changes in prostate cancer cell lines, based on the GSE259218, GSE200204, GSE80657, GSE53012, and GSE78245 datasets from GEO database. Data in (**D**) were not normally distributed, and analyzed using Mann-Whitney U test. Data in (**B**, **C**, **E-I**) were normally distributed and analyzed by Student’s t test. (**J**, **K**) qRT-PCR for the mRNA expression change of FOXD1 in PC-3 and 22Rv1 cells 24, 48, and 72 h after treatment with hypoxia. (**L**, **M**) Western blotting for the protein expression change of FOXD1 in PC-3 and 22Rv1 cells 24, 48, and 72 h after treatment with hypoxia. Data are expressed as the means ± SD, and were analyzed by one-way ANOVA followed by Dunnett’s post hoc test. ***p < 0.001 vs. cells under normoxia conditions

The datasets of GSE259218, GSE200204, GSE80657, and GSE53012 were used to analyze the hypoxia-related genes in PC-3 cells, and a total of 60 genes were identified and designated as Set “I”. GSE259218 and GSE80657 were used for the analysis of hypoxia-related genes in 22Rv1 cells, and 2215 candidate genes were identified and designated as Set “II”. In addition, 1515 genes (designated as Set “III”) in DU145 cells and 368 genes (designated as Set “IV”) in LNCaP cells were identified in GSE80657 and GSE78245, respectively. The Sets I, II, III, and IV were then further analyzed to obtain the common hypoxia-related genes, and 6 common genes (BNIP3L, C4orf3, P4HA2, RAB31, HK2, and FOXD1) were obtained (Fig. 1A). The mRNA expression levels of BNIP3L, C4orf3, and P4HA2 did not change statistically in the normal group and the cancer group (Supplementary Fig. S1A-C). RAB31 mRNA expression level was decreased in cancer group compared to that in normal group (Supplementary Fig. S1D), but after hypoxic treatment, the mRNA level of RAB31 increased in some prostate cancer cell lines while decreased in others (Table 1). The expression levels of HK2 and FOXD1 mRNA were both increased in cancer group compared to those in normal group (Supplementary Fig. S1E, F), and HK2 (Table 2) and FOXD1 (Fig. 1B-I) mRNA levels were both increased after hypoxic treatment in prostate cancer PC-3, 22Rv1, DU145, and LNCaP cells. Since the roles of hypoxia-induced HK2 in prostate cancer cells have been widely reported [23–25], we mainly focused on FOXD1, and HK2 was only detected as a key glycolytic enzyme in the following experiments. Next, we investigated the expression changes of FOXD1 in PC-3 and 22Rv1 cells under hypoxia or normoxia conditions. qRT-PCR demonstrated that under hypoxia conditions for 24, 48 and 72 h, the mRNA levels of FOXD1 were increased in both PC-3 (Fig. 1J) and 22Rv1 (Fig. 1K) cells. Similarly, western blotting showed that the protein expression levels of FOXD1 were increased under hypoxia conditions in both PC-3 (Fig. 1L) and 22Rv1 (Fig. 1M) cells.

**Table 1:** The changes in RAB31 mRNA expression in different prostate cancer cell lines after hypoxia treatment based on the GSE datasets from GEO database

GSE	Cell line	Adjusted *p* value	log(Fold change)
GSE200204	PC-3	0.047123	-0.7954108
GSE259218	PC-3	9.62E-04	-0.2495463
GSE259218	22Rv1	0.00583887	1.54
GSE53012	PC-3	0.0011368	-1.43
GSE78245	LNCaP	0.019687	0.837157
GSE80657	DU145	0.00060277	1.24417867
GSE80657	PC-3	0.03929992	-0.355
GSE80657	22Rv1	5.41E-12	1.39

**Table 2:** The changes in HK2 mRNA expression in different prostate cancer cell lines after hypoxia treatment based on the GSE datasets from GEO database

GSE	Cell line	Adjusted *p* value	log(Fold change)
GSE200204	PC-3	0.033324	0.9432925
GSE259218	PC-3	1.90E-07	0.90661946
GSE259218	22Rv1	0.00294111	1.95
GSE53012	PC-3	0.0006669	1.78
GSE78245	LNCaP	0.009238	0.8905897
GSE80657	DU145	0.03047258	0.30496608
GSE80657	PC-3	0.00028167	1.19
GSE80657	22Rv1	9.42E-19	2.19

### FOXD1 is upregulated in prostate cancer

**Fig. 2:**
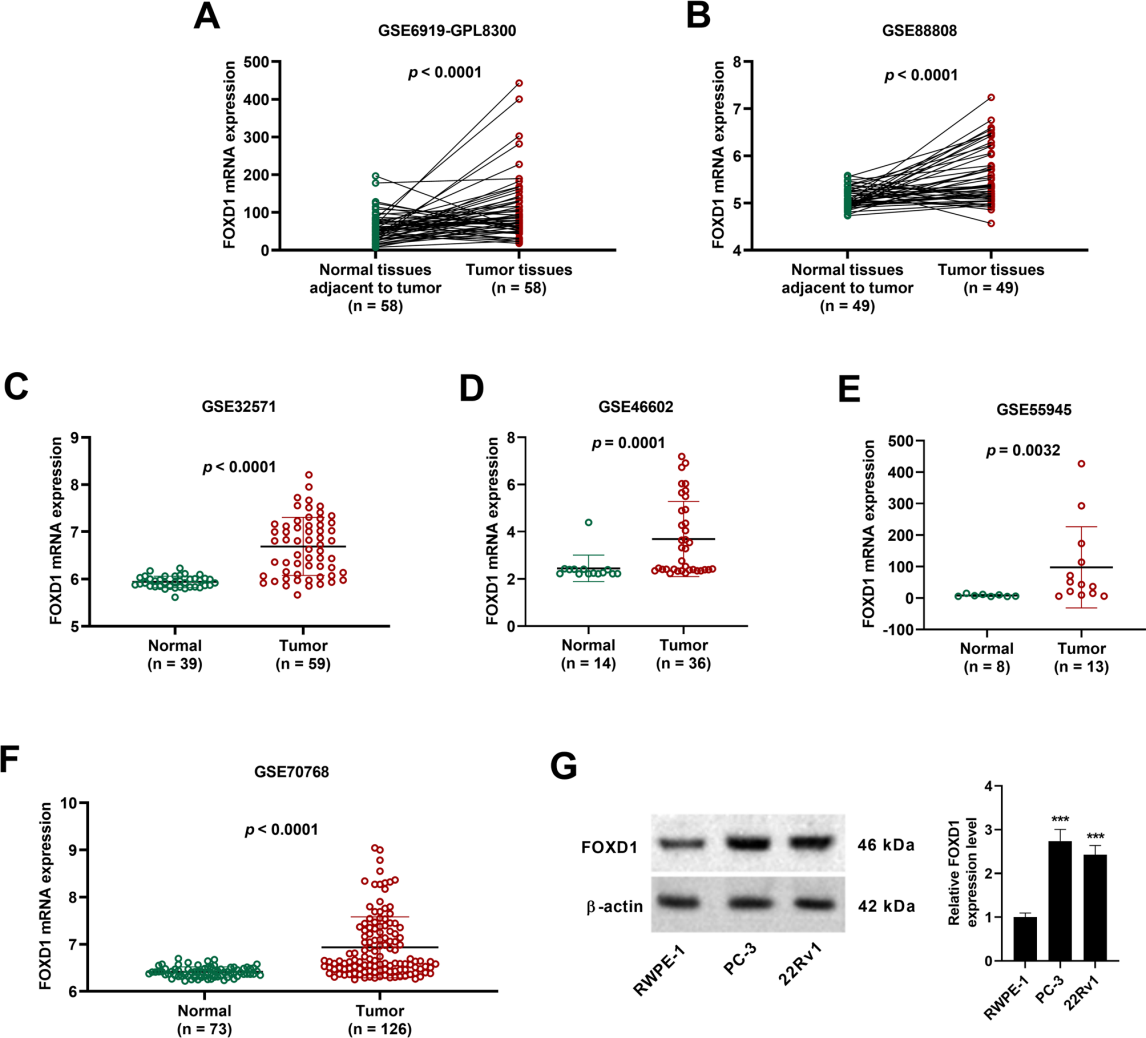
FOXD1 expression is upregulated in prostate cancer. (**A-F**) Analysis of FOXD1 mRNA changes in prostate cancer patients based on the GEO databases. Data in (**A**, **B**) were not normally distributed, and analyzed using Wilcoxon signed-rank test. Data in (**C-F**) were not normally distributed, and analyzed by Mann-Whitney U test. (**G**) Western blotting for the expression level of FOXD1 in PC-3 and 22Rv1 cells under normoxia conditions. Data are expressed as the means ± SD, and were analyzed using one-way ANOVA followed by Dunnett’s post hoc test. ***p < 0.001 vs. RWPE-1 cells

As compared to normal tissues adjacent to tumor, the mRNA levels of FOXD1 were significantly increased in tumor tissues (Fig. 2A, B). Besides, FOXD1 mRNA levels were found to be increased in prostate cancer patients, as compared to normal individuals (Fig. 2C-F). Our western blotting experiments proved that protein level of FOXD1 was increased in PC-3 and 22Rv1 cells when compared with that in human prostatic epithelial cell line RWPE-1 (Fig. 2G).

### FOXD1 is a tumor-promoter in prostate cancer cells

**Fig. 3:**
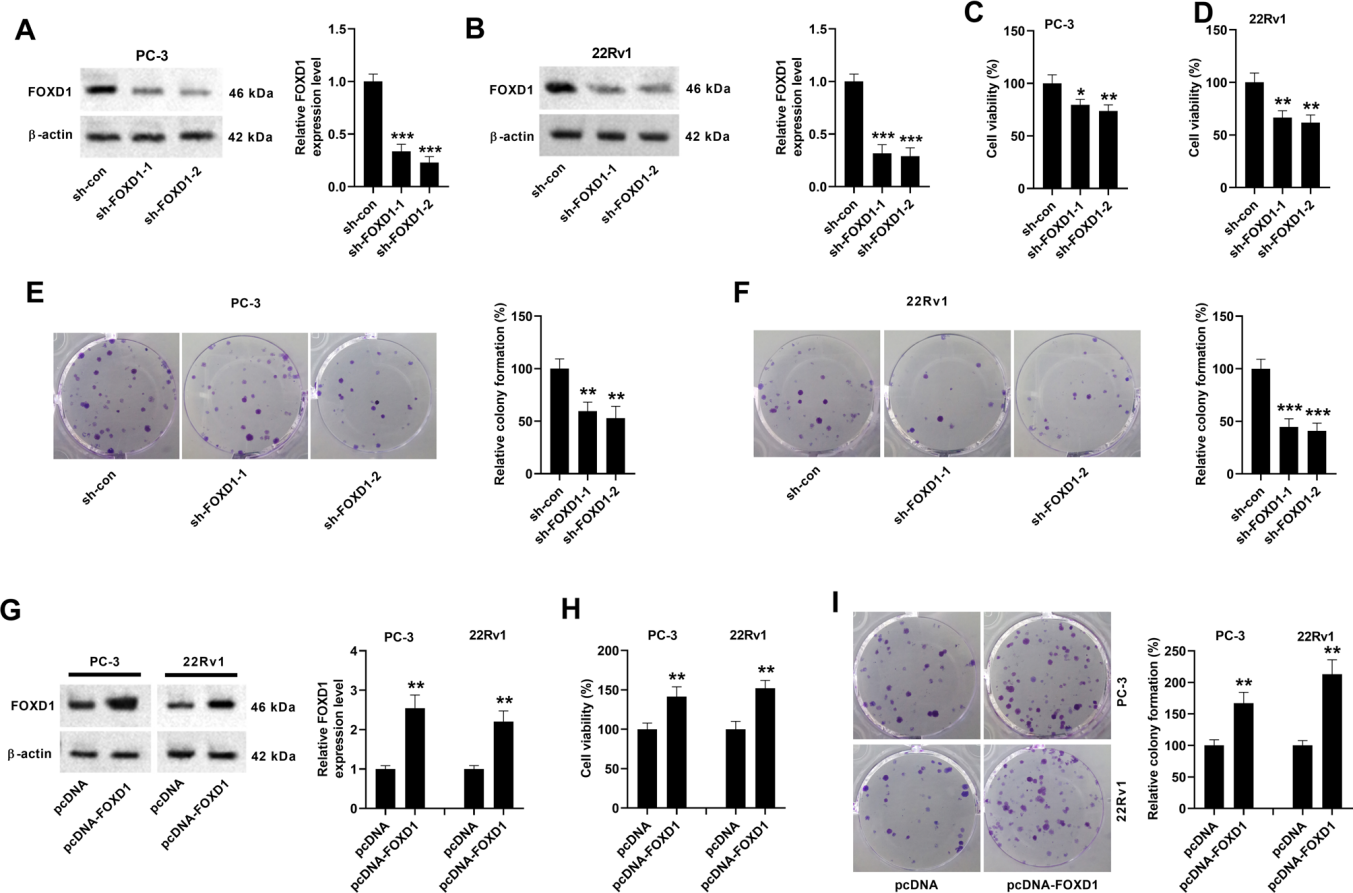
FOXD1 regulates cell viability and colony formation of prostate cancer cells. (**A**, **B**) FOXD1-knockdown cells were constructed through transfection with sh-FOXD1-1 or sh-FOXD1-2. Western blotting showed that FOXD1 expression was decreased in PC-3 and 22Rv1 cells 72 h after transfection with sh-FOXD1-1 or sh-FOXD1-2. Data are expressed as the means ± SD, and were analyzed using one-way ANOVA followed by Dunnett’s post hoc test. ***p < 0.001 vs. sh-con group. (**C**, **D**) Knockdown of FOXD1 decreased the viability of PC-3 and 22Rv1 cells. Data are expressed as the means ± SEM, and were analyzed using one-way ANOVA followed by Dunnett’s post hoc test. *p < 0.05, **p < 0.01 vs. sh-con group. (**E**, **F**) Knockdown of FOXD1 inhibited the colony formation of PC-3 and 22Rv1 cells. Data are expressed as the means ± SD, and were analyzed using one-way ANOVA followed by Dunnett’s post hoc test. **p < 0.01, ***p < 0.001 vs. sh-con group. (**G**) FOXD1-overexpression cells were also established through transfection with pcDNA-FOXD1. Western blotting was conducted 72 h after transfection. Data are expressed as the means ± SD, and were analyzed by Student’s t test. **p < 0.01 vs. pcDNA group. (**H**) Overexpression of FOXD1 elevated the viability of PC-3 and 22Rv1 cells. Data are expressed as the means ± SEM, and were analyzed by Student’s t test. **p < 0.01 vs. pcDNA group. (**I**) Overexpression of FOXD1 elevated the colony formation of PC-3 and 22Rv1 cells. Data are expressed as the means ± SD, and were analyzed by Student’s t test. **p < 0.01 vs. pcDNA group

To explore the roles of FOXD1 in prostate cancer cells, FOXD1-knockdown cells were constructed through transfection with sh-FOXD1-1 or sh-FOXD1-2. Western blotting showed that FOXD1 expression was decreased in PC-3 and 22Rv1 cells after sh-FOXD1-1 or sh-FOXD1-2 transfection (Fig. 3A, B). Knockdown of FOXD1 decreased the viability of PC-3 and 22Rv1 cells (Fig. 2C, D). Colony formation of PC-3 or 22Rv1 cells was inhibited by transfection with sh-FOXD1-1 or sh-FOXD1-2 (Fig. 3E, F). Additionally, FOXD1-overexpression cells were also established through transfection with pcDNA-FOXD1 (Fig. 3G). Overexpression of FOXD1 enhanced the viability and colony formation of PC-3 or 22Rv1 cells (Fig. 3H, I). These findings indicate that FOXD1 might be a tumor-promoter in prostate cancer.

### FOXD1 knockdown inhibits hypoxia-induced prostate cancer cell growth

**Fig. 4:**
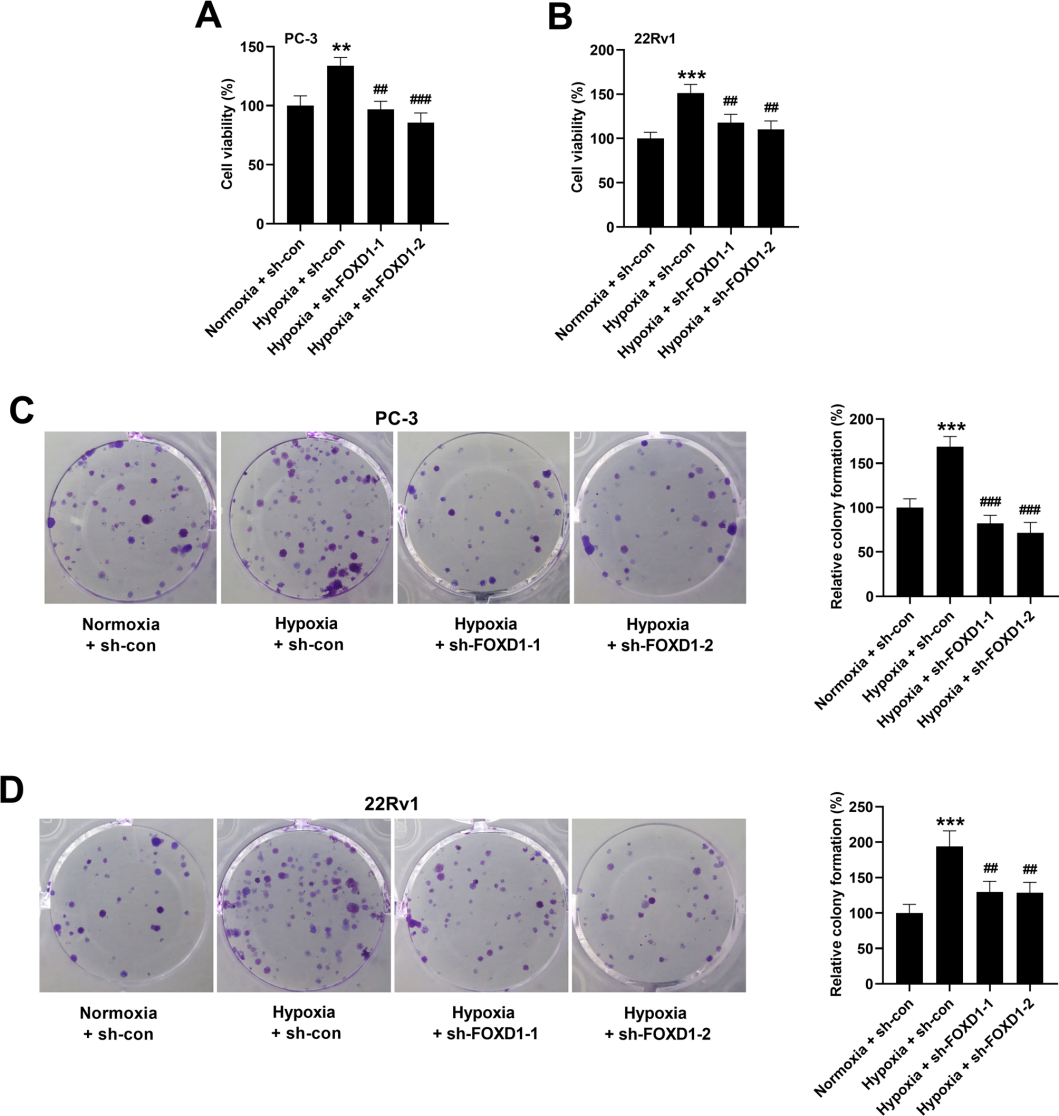
FOXD1 knockdown inhibits hypoxia-induced cell viability and colony formation in prostate cancer cells. (**A**, **B**) Cells were transfected with sh-con, sh-FOXD1-1, or sh-FOXD1-2, and then cultured under normoxia or hypoxia conditions for 72 h. Role of FOXD1 in regulating prostate cancer cell viability was evaluated using CCK-8 assay. Data are expressed as the means ± SEM, and were analyzed using one-way ANOVA followed by Tukey’s post hoc test. (**C**, **D**) Cells were transfected with sh-con, sh-FOXD1-1, or sh-FOXD1-2, and then cultured under normoxia or hypoxia conditions for 10 d. Effect of FOXD1 on colony formation of PC-3 and 22Rv1 cells was then evaluated. Data are expressed as the means ± SD, and were analyzed using one-way ANOVA followed by Tukey’s post hoc test. **p < 0.01, ***p < 0.001 vs. cells transfected with sh-con and cultured under normoxia conditions. ^##^*p* < 0.01, ^###^*p* < 0.001 vs. cells transfected with sh-con and cultured under hypoxia conditions

Subsequently, the roles of FOXD1 in regulating prostate cancer cell growth under hypoxia conditions were evaluated. As indicated in Fig. 4A, B, hypoxia induced the viability in both PC-3 and 22Rv1 cells, which were attenuated by sh-FOXD1-1 or sh-FOXD1-2. Besides, colony formation of PC-3 or 22Rv1 cells was increased after hypoxia induction. However, FOXD1 knockdown prevented hypoxia-induced colony formation (Fig. 4C, D).

### FOXD1 knockdown inhibits hypoxia-induced glycolysis

**Fig. 5:**
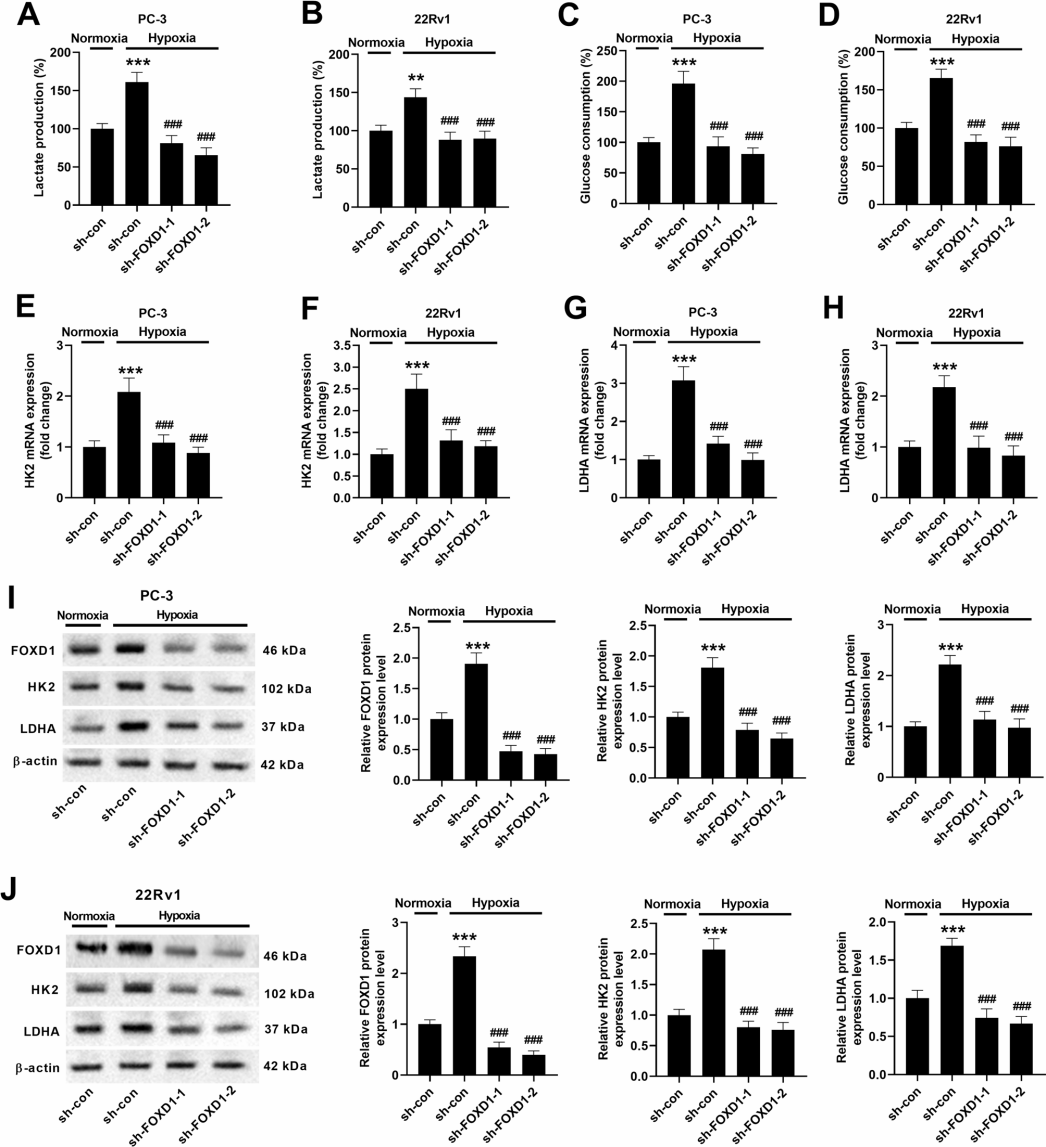
FOXD1 knockdown inhibits hypoxia-induced glycolysis in prostate cancer cells. Cells were transfected with sh-con, sh-FOXD1-1, or sh-FOXD1-2, and then cultured under normoxia or hypoxia conditions for 72 h. (**A-D**) Lactate production and glucose consumption were determined. Data are expressed as the means ± SEM, and were analyzed using one-way ANOVA followed by Tukey’s post hoc test. (**E-H**) The mRNA expression levels of HK-2 and LDHA in PC-3 and 22Rv1 cells were detected by qRT-PCR. Data are expressed as the means ± SD, and were analyzed using one-way ANOVA followed by Tukey’s post hoc test. ***p < 0.001 vs. cells transfected with sh-con and cultured under normoxia conditions. ^###^*p* < 0.001 vs. cells transfected with sh-con and cultured under hypoxia conditions. (**I**, **J**) The protein expression levels of FOXD1, HK-2, and LDHA in PC-3 and 22Rv1 cells were detected by western blotting. Data are expressed as the means ± SD, and were analyzed using one-way ANOVA followed by Tukey’s post hoc test. ***p < 0.001 vs. cells transfected with sh-con and cultured under normoxia conditions. ^###^*p* < 0.001 vs. cells transfected with sh-con and cultured under hypoxia conditions

After hypoxia induction, the lactate production and glucose consumption in PC-3 and 22Rv1 cells were increased, while FOXD1 knockdown reversed the increase in lactate production and glucose consumption (Fig. 5A-D). Under hypoxia conditions, the mRNA expression levels of glycolytic enzymes, including HK-2 and LDHA, were elevated, while FOXD1 knockdown inhibited this elevation (Fig. 5E-H). Moreover, the protein expression levels of FOXD1, HK-2, and LDHA were increased under hypoxia conditions. FOXD1 knockdown decreased these protein levels in PC-3 and 22Rv1 cells under hypoxia conditions (Fig. 5I, J).

### Inhibition of glycolysis reverses the effect of FOXD1 overexpression on hypoxia-induced prostate cancer cell growth

**Fig. 6:**
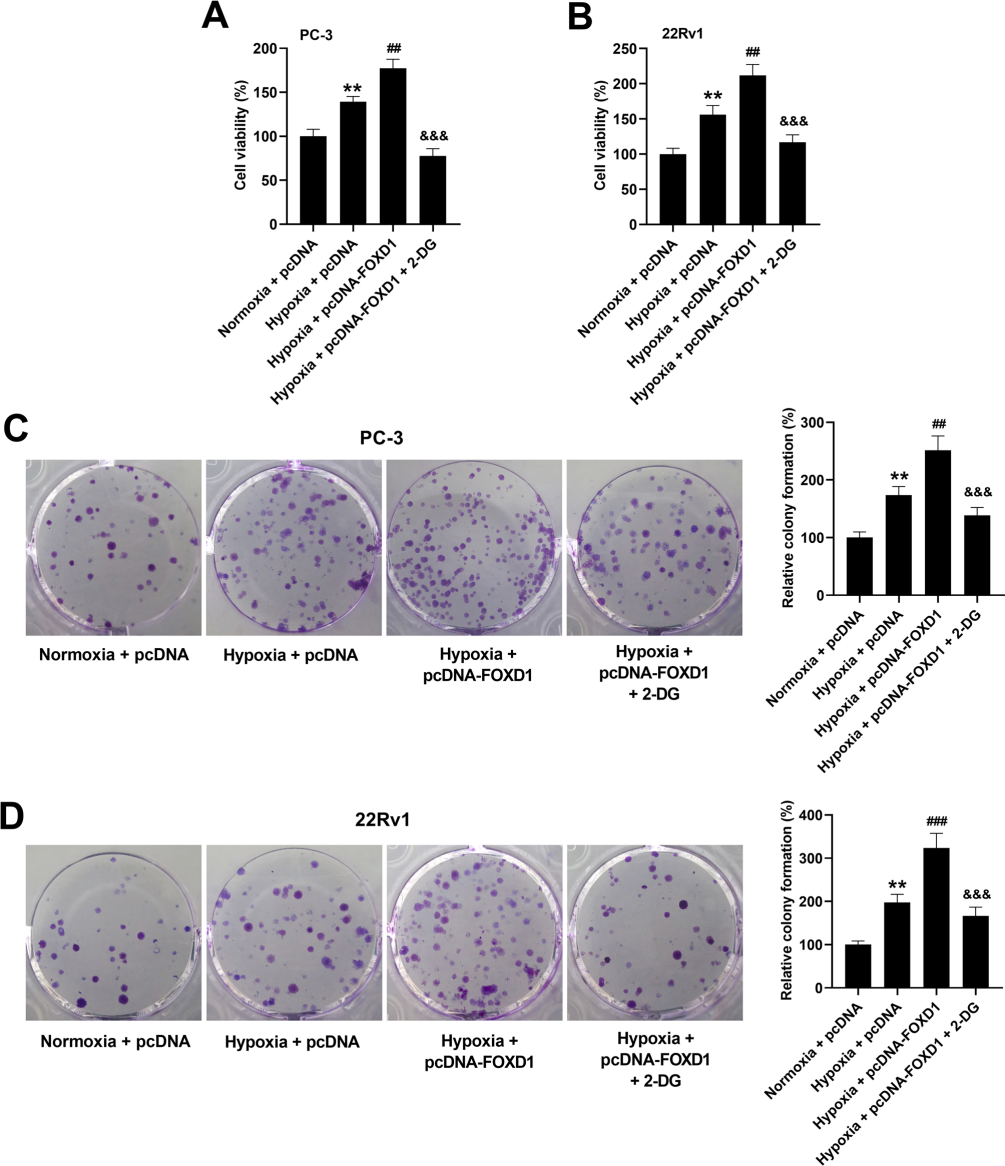
Glycolysis inhibitor reverses the inductive effects of FOXD1 overexpression on the viability and colony formation of prostate cancer cells under hypoxia conditions. (**A**, **B**) Cells were transfected with pcDNA or pcDNA-FOXD1, and then cultured in the presence or absence of 5 mM 2-deoxyglucose (2-DG) under normoxia or hypoxia conditions for 72 h. Cell viability was evaluated using CCK-8 assay. Data are expressed as the means ± SEM, and were analyzed using one-way ANOVA followed by Tukey’s post hoc test. (**C**, **D**) Cells were transfected with pcDNA or pcDNA-FOXD1, and then cultured in the presence or absence of 5 mM 2-deoxyglucose (2-DG) under normoxia or hypoxia conditions for 10 d. Colony formation of PC-3 and 22Rv1 cells was then evaluated. Data are expressed as the means ± SD, and were analyzed using one-way ANOVA followed by Tukey’s post hoc test. **p < 0.01 vs. cells transfected with pcDNA plasmid and cultured under normoxia conditions. ^##^*p* < 0.01, ^###^*p* < 0.001 vs. cells transfected with pcDNA plasmid and cultured under hypoxia conditions. ^&&&^*p* < 0.001 vs. cells transfected with pcDNA plasmid and cultured under hypoxia conditions

To confirm the role of glycolysis in mediating the effects of FOXD1, cells were treated with 2-DG to inhibit glycolysis process. As shown in Fig. 6A, B, FOXD1 overexpression strengthen hypoxia-induced increase in the viability of PC-3 and 22Rv1 cells. However, 2-DG treatment prevented the inductive effect of FOXD1 overexpression on cell viability. Besides, hypoxia-induced increase in colony formation was enhanced by FOXD1 overexpression, which could be reversed by 2-DG treatment (Fig. 6C, D). The results indicate that glycolysis mediates the effects of FOXD1 on prostate cancer cell growth under hypoxia conditions.

### FOXD1 overexpression promotes hypoxia-induced glycolysis

**Fig. 7:**
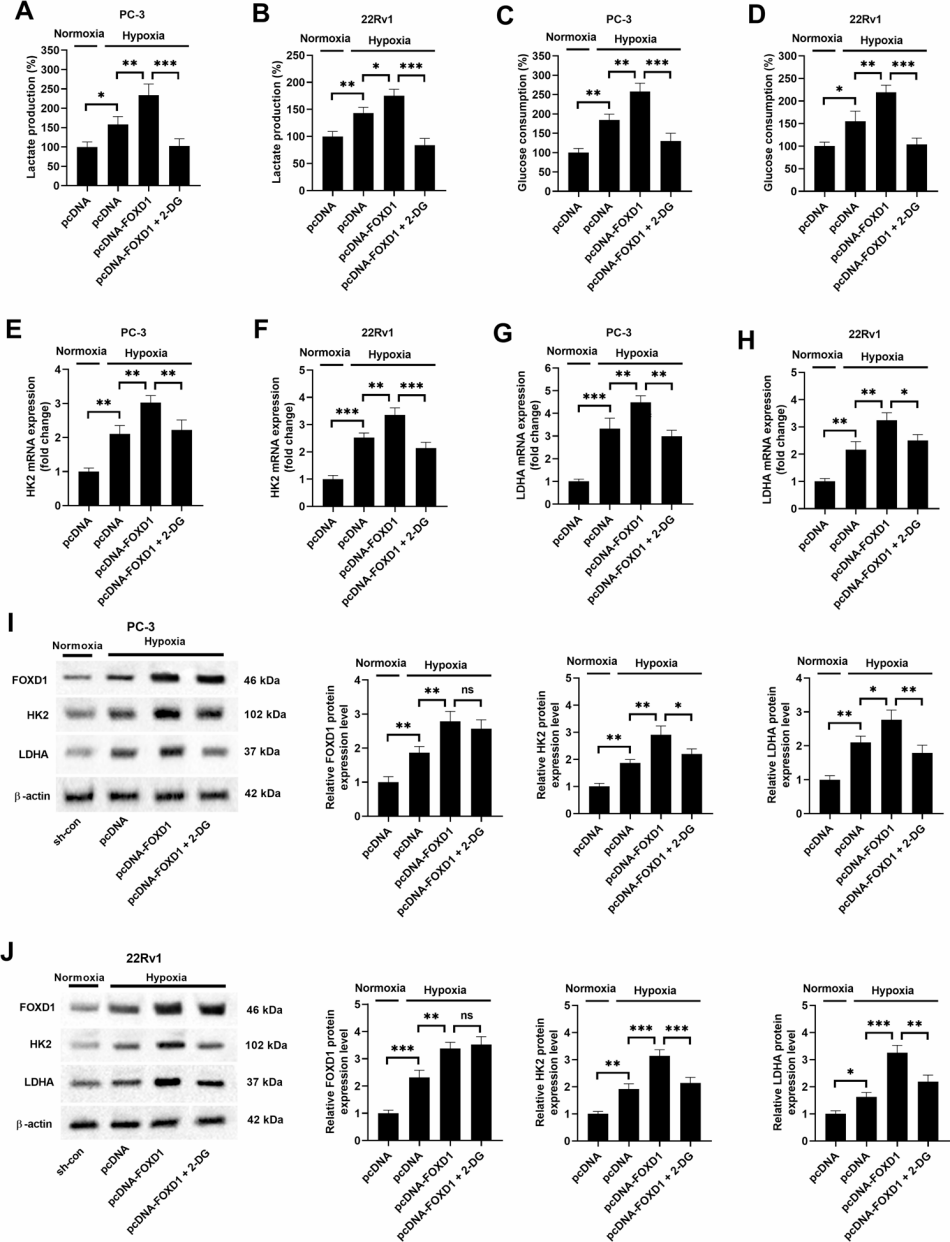
FOXD1 overexpression promotes hypoxia-induced glycolysis. Cells were transfected with pcDNA or pcDNA-FOXD1, and then cultured in the presence or absence of 5 mM 2-deoxyglucose (2-DG) under normoxia or hypoxia conditions for 72 h. (**A-D**) Lactate production and glucose consumption were determined. Data are expressed as the means ± SEM, and were analyzed using one-way ANOVA followed by Tukey’s post hoc test. (**E-H**) The mRNA expression levels of HK-2 and LDHA in PC-3 and 22Rv1 cells were detected by qRT-PCR. Data are expressed as the means ± SD, and were analyzed using one-way ANOVA followed by Tukey’s post hoc test. (**I**, **J**) The protein expression levels of FOXD1, HK-2, and LDHA in PC-3 and 22Rv1 cells were detected by western blotting. Data are expressed as the means ± SD, and were analyzed using one-way ANOVA followed by Tukey’s post hoc test. *p < 0.05, **p < 0.01, ***p < 0.001. ns, no statistical significance

After hypoxia treatment, the lactate production and glucose consumption in PC-3 and 22Rv1 cells were increased. FOXD1 overexpression enhanced the effect of hypoxia on these indexes, which was inhibited by 2-DG treatment (Fig. 7A-D). The mRNA expression levels of HK-2 and LDHA were elevated under hypoxia conditions. FOXD1 overexpression further promoted this elevation, while 2-DG counteracted the effect of FOXD1 (Fig. 7E-H). Moreover, FOXD1 overexpression boosted FOXD1 protein levels, and 2-DG did not affect FOXD1 levels. Hypoxia increased the expression of HK-2 and LDHA, and this effect was amplified by FOXD1 overexpression. Notably, 2-DG suppressed the FOXD1-mediated enhancement of HK-2 and LDHA protein levels under hypoxia conditions (Fig. 7I, J). The results indicate that FOXD1 overexpression promotes hypoxia-induced glycolysis.

## Discussion

The expression status and roles of FOXD1 in many types of cancer have been reported. For example, FOXD1 upregulation is associated with poor outcome in patients with basal-like breast cancer, and FOXD1 maintains tumor-promoting enhancer-gene programs [26]. High level of FOXD1 is associated with poor prognosis of pancreatic cancer and facilitates the proliferation, invasion, and metastasis of pancreatic cancer cells [27]. FOXD1 upregulation promotes gastric cancer cell proliferation, motility, and cisplatin resistance [28]. FOXD1 promotes colorectal cancer cell stemness and enhances chemotherapy resistance [29]. It has been reported that FOXD1 is extensively detected in oral cancer tissues and associated with poor outcome in patients receiving irradiation since it may lessen anti-cancer radiotherapy effectiveness and immune surveillance [30]. However, the roles of FOXD1 in prostate cancer remain largely unknown. Our experiments proved that protein level of FOXD1 was increased in prostate cancer PC-3 and 22Rv1 cells. Knockdown of FOXD1 decreased the viability and colony formation of PC-3 and 22Rv1 cells, while overexpression of FOXD1 exhibited opposite effects, implying that FOXD1 might be a tumor-promoter in prostate cancer cells.

Oxygen homeostasis is essential for mammalian cells to ensure the physiological functions [31, 32]. When the oxygen concentration decreases, called hypoxia, a series of downstream responses are activated mainly including hypoxia-inducible factor signaling, autophagy, and other hypoxia stress [33]. These hypoxic responses are linked to a variety of processes, such as cell growth/death, cell proliferation, immune responses, glycolysis, metabolic adaption, and tumorigenesis [34]. It is evident that hypoxia is an important and common feature of solid malignancies [35]. Tumor cells response to hypoxia may undergo reoxygenation, and they may convert to a more aggressive phenotype during the hypoxia-reoxygenation cycle [36]. In addition to affecting cancer cells, intratumoral hypoxia also influence the stromal cells within the tumor, most notably resulting in the function loss of immune cells [37, 38]. Recent studies have documented that the increased activity of hypoxia-inducible factor drives immune evasion [39]. A large number of studies have found that pharmacologic inhibiting hypoxic signaling exerts positive effects on the therapy of cancers including prostate cancer [40]. Despite FOXD1 has been found to be involved in several types of cancer, its role in mediating prostate cancer cells under hypoxia conditions is unclear. Our results showed that FOXD1 expression was upregulated in prostate cancer cell lines under hypoxia conditions compared with cells under normoxia conditions, based on the data analysis of GEO databases. Further results of western blotting indicated that the expression level of FOXD1 was increased under hypoxia conditions in both PC-3 and 22Rv1 cells. FOXD1 knockdown inhibited hypoxia-induced prostate cancer cell growth. These findings implied that FOXD1 might be a hypoxia-related gene in prostate cancer.

It is reported that HIF-1α plays a vital part in the cellular response to hypoxia through regulating the expression of numerous genes participating in adaptive processes that enable cell survival under low oxygen conditions [41]. As shown in Supplementary Fig. S2A, B, the expression levels of HIF-1α were increased under hypoxia conditions for 12 and 24 h. However, when exposed to hypoxia for 48 and 72 h, the levels of HIF-1α decreased to a level equivalent to that under normoxia conditions. According to Fig. 1L, M, compared with the normoxic conditions, the expression levels of FOXD1 were increased during the period from 24 to 72 h under hypoxia conditions. Based on the above results, we speculated that FOXD1 is not a target gene of HIF-1α. It has been shown that hypoxia induces activation of the Akt pathway in tumors [42, 43]. Furthermore, the Akt pathway has been reported to regulate FOXD1 expression in tumors [44]. Thus, we inferred that hypoxia upregulates FOXD1 expression by activating the Akt pathway in prostate cancer.

Glycolysis is a universal metabolism of glucose in the living cells, which describes an anaerobic conversion of glucose into two molecules of pyruvate [45]. The process takes place in the cytoplasm and results in the sufficient generation of adenosine triphosphate through an oxygen-independent glycolytic pathway [46]. Within the tumor microenvironment, hypoxia accelerates the rate of glycolysis, and then promotes the proliferation of cancer cells and contributes to the immunoevasive tumor phenotype [47]. Besides, highly glycolytic tumor cells deprive the adequate glucose supply of immune cells and thus suppress T cell activity [48, 49]. The end product of glycolysis lactate in the tumor microenvironment also reduces the activity of immune cells, thus to potentiate the immunosuppressive effects [50, 51]. Taken together, inhibition of glycolysis in cancer cells is considered as a novel approach for anti-tumor therapies. A previous study has shown that under hypoxia conditions, HK2, LDHA, and glucose transporter 1 (GLUT1) are upregulated in prostate cancer cells, and brusatol treatment inhibits glycolysis by decreasing the expression of these glycolysis-related enzymes [52]. Similarly, knockdown of phospholipase C-ε suppresses hypoxia-induced increase in the expression of HK2, LDHA, and GLUT1 in some types of prostate cancer cell lines [25]. In this study, we found that FOXD1 knockdown inhibited hypoxia-induced glycolysis by decreasing HK2 and LDHA expression in PC-3 and 22Rv1 cells. Inhibition of glycolysis by 2-DG reversed the effect of FOXD1 overexpression on hypoxia-induced prostate cancer cell growth, implying the FOXD1 exerted its roles via regulating glycolysis. Previous studies have shown that FOXD1 in tumors affects the glycolysis process by regulating multiple glycolysis-related enzymes. For example, FOXD1 enhances the aerobic glycolysis process by increasing HK2, LDHA, and GLUT1 expression in breast cancer cells [53]. FOXD1 accelerates aerobic glycolysis by increasing GLUT1 expression, ultimately promoting the malignant behaviors of pancreatic cancer cells [27]. GLUT1 is widely expressed in normal tissue and shows higher expression levels in numerous tumors [54]. In several in vitro and in vivo tumor models, GLUT1 is upregulated by hypoxia, and it plays a significant role in promoting tumor growth [55]. Additionally, GLUT1 has been demonstrated to be a candidate marker of hypoxia in prostate cancer [56]. Based on the above reports, it can be speculated that under hypoxic conditions, FOXD1 can also affect glycolysis by regulating GLUT1 expression in prostate cancer cells. However, this needs to be verified by experiments.

The findings of this study hold substantial clinical relevance for prostate cancer. According to the databases, FOXD1 is significantly upregulated in prostate cancer tissues and further induced by intratumoral hypoxia. Clinically, FOXD1 expression could serve as a possible prognostic marker. Moreover, intratumoral hypoxia is a major barrier to prostate cancer therapy, and it reduces the efficacy of radiotherapy and chemotherapy. Clinically, FOXD1 inhibition could be combined with existing therapies to improve outcomes. Future studies should validate the prognostic and predictive values of FOXD1 in large clinical cohorts and explore the safety and efficacy of FOXD1-targeted therapies in preclinical and clinical models. This study has several limitations. Firstly, the study only used two prostate cancer cell lines (PC-3 and 22Rv1) for in vitro experiments, lacking verification in animal xenograft models. Secondly, the mechanism by which hypoxia upregulates FOXD1 expression was only inferred to possibly involve the Akt pathway based on existing literature, without direct experimental validation to confirm this regulatory relationship. Moreover, there was no analysis of the clinical correlation between FOXD1 expression and glycolysis-related molecule levels (e.g., HK-2 and LDHA) in clinical prostate cancer samples, which weakens the clinical translational value of the study’s findings.

**Fig. 8:**
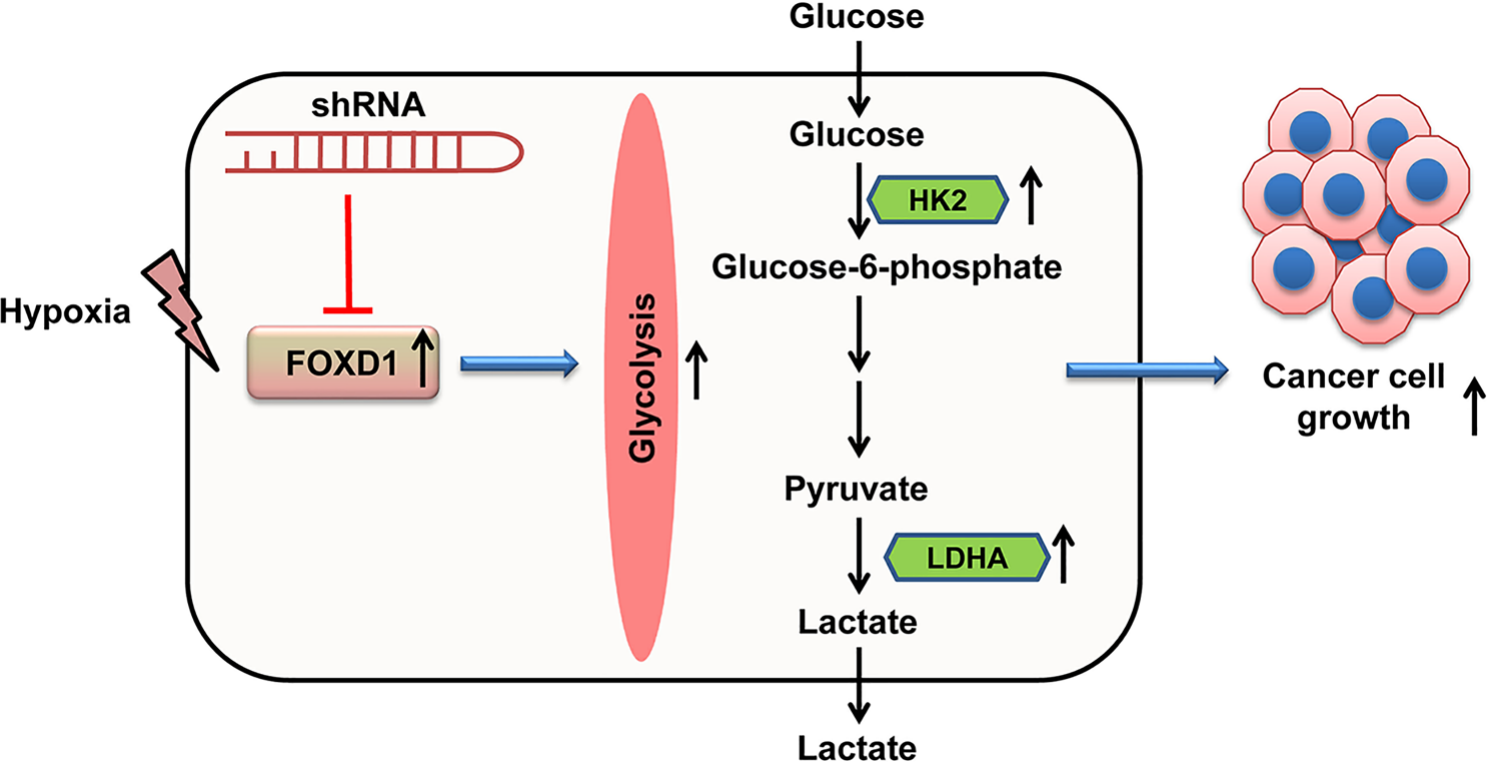
The schematic illustration of role of FOXD1 in glycolysis and prostate cancer cell growth under hypoxia conditions. Hypoxia induces FOXD1 expression, which leads to increased glycolysis, and ultimately accelerating prostate cancer cell growth. However, shRNA targeting FOXD1 inhibits FOXD1 expression, and then decreases glycolysis, resulting in suppression of prostate cancer cell growth

In conclusion, this study showed that FOXD1 was identified as a hypoxia-related gene and tumor promoter in prostate cancer. FOXD1 knockdown inhibited hypoxia-induced prostate cancer cell growth, which was mediated by glycolysis (Fig. 8). Therefore, FOXD1 might be a potential therapeutic target for the treatment of prostate cancer.

## Supplementary Information

Below is the link to the electronic supplementary material.

Supplementary Material 1

Supplementary Material 2

## Data Availability

Human data analyzed in this study are available at the GEPIA database (http://gepia.cancer-pku.cn/) and GEO database (https://www.ncbi.nlm.nih.gov/geo/). Cellular experimental data were included in the manuscript or supplementary files.
