# Supplementary material for: FOXD1, a hypoxia-related gene, accelerates prostate cancer cell growth by increasing glycolysis under hypoxia conditions
Source: BMC Biotechnol. 2025 Nov 10;25:123. doi: 10.1186/s12896-025-01061-6 (PMC12604200; doi:10.1186/s12896-025-01061-6)
Supplement: Supplementary file 1 — Supplementary Material 1 [file 12896_2025_1061_MOESM1_ESM.docx]

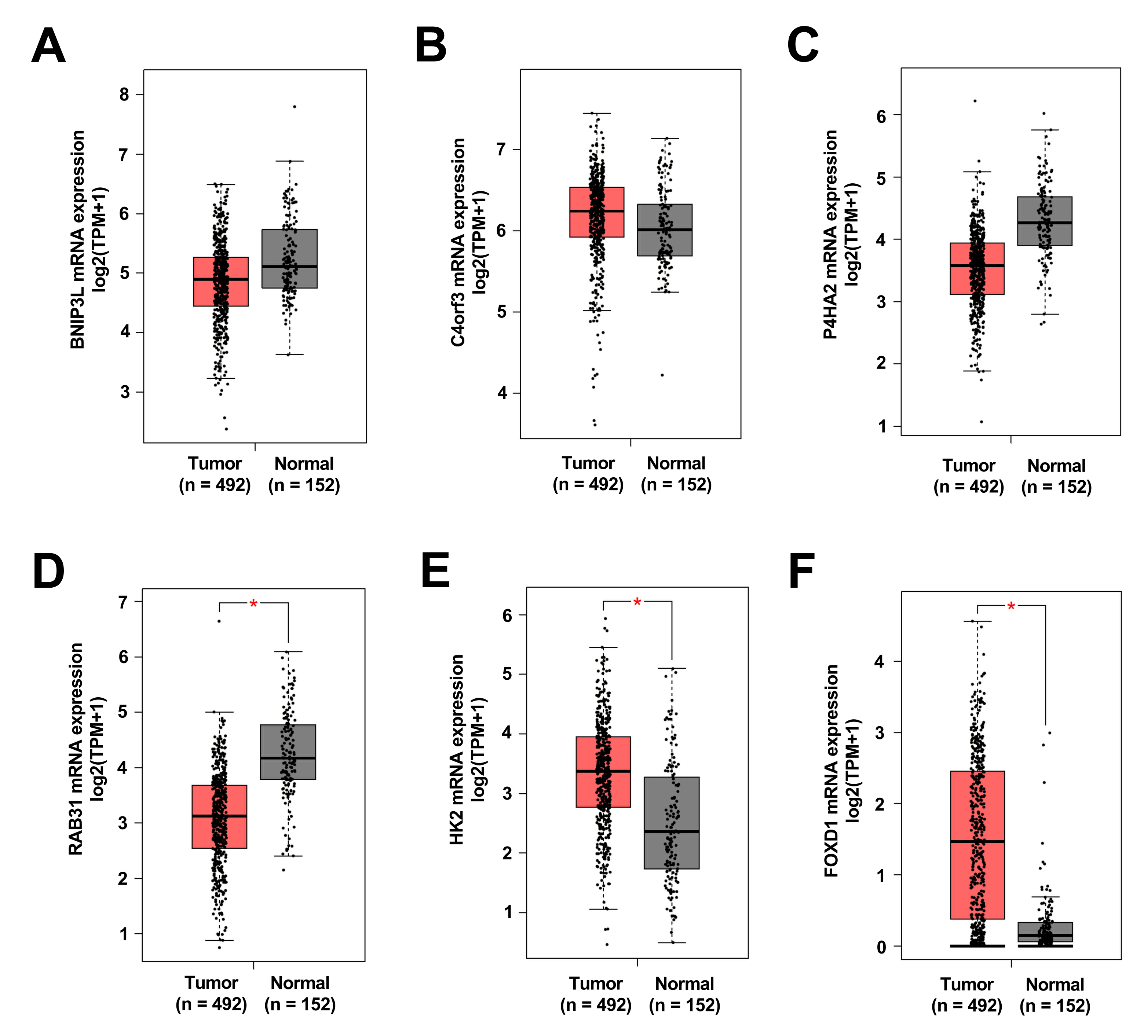


**Supplementary Figure S1.** Analysis of BNIP3L, C4orf3, P4HA2, RAB31, HK2, and FOXD1 mRNA changes in prostate cancer patients and tumor tissues from the TCGA databases based on GEPIA online tool. The mRNA expression values of BNIP3L (A), C4orf3 (B), P4HA2 (C), RAB31 (D), HK2 (E), and FOXD1 (F) in 492 prostate cancer tissues and 152 control tissues were all obtained from GEPIA online tool. “*” indicates statistical significance.


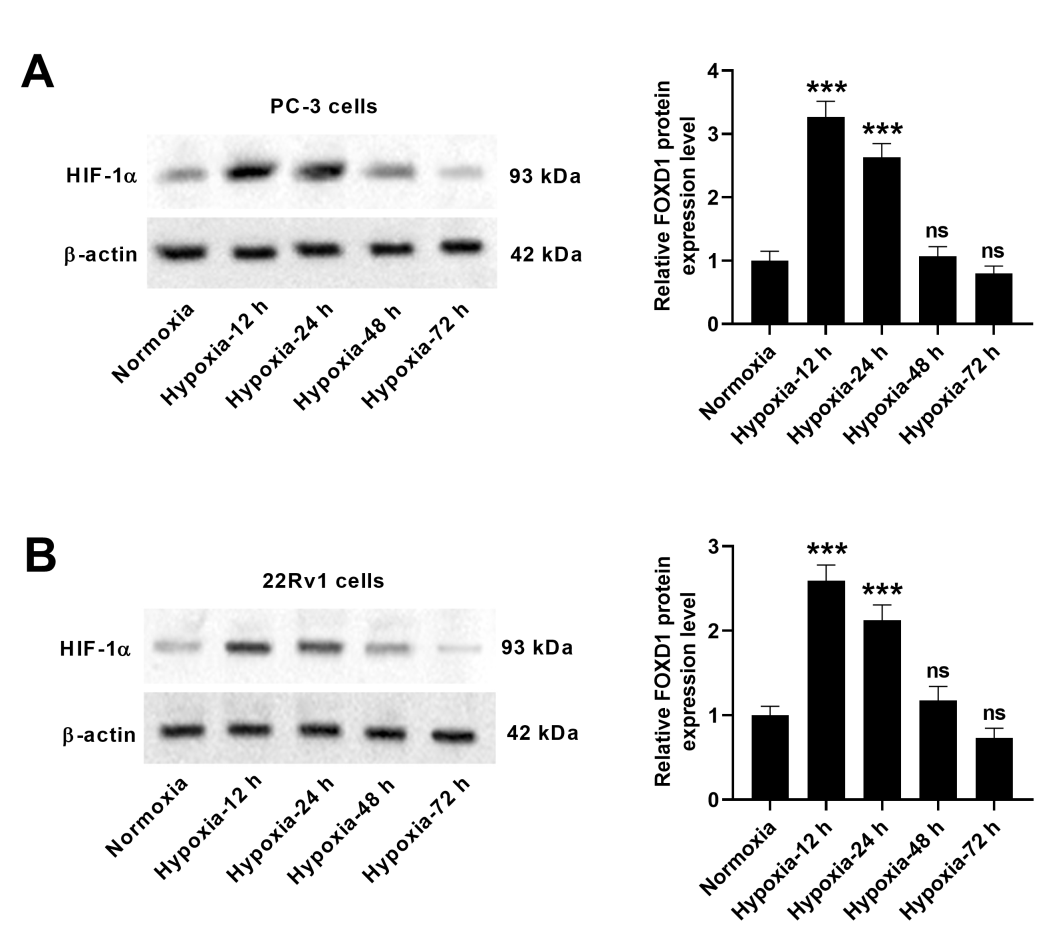


**Supplementary Figure S2.** The expression status of HIF-1α in prostate cancer in response to hypoxia. (A, B) Western blotting for the protein expression of HIF-1α in PC-3 and 22Rv1 cells 12, 24, 48, and 72 h after treatment with hypoxia. Data are expressed as the means ± SD, and were analyzed by one-way ANOVA followed by Dunnett’s post hoc test. ****p* < 0.001 vs. cells under normoxia conditions. ns, no statistical significance.
