# Supplementary figures and images for: FOXD1, a hypoxia-related gene, accelerates prostate cancer cell growth by increasing glycolysis under hypoxia conditions
Source: BMC Biotechnol. 2025 Nov 10;25:123. doi: 10.1186/s12896-025-01061-6 (PMC12604200; doi:10.1186/s12896-025-01061-6)

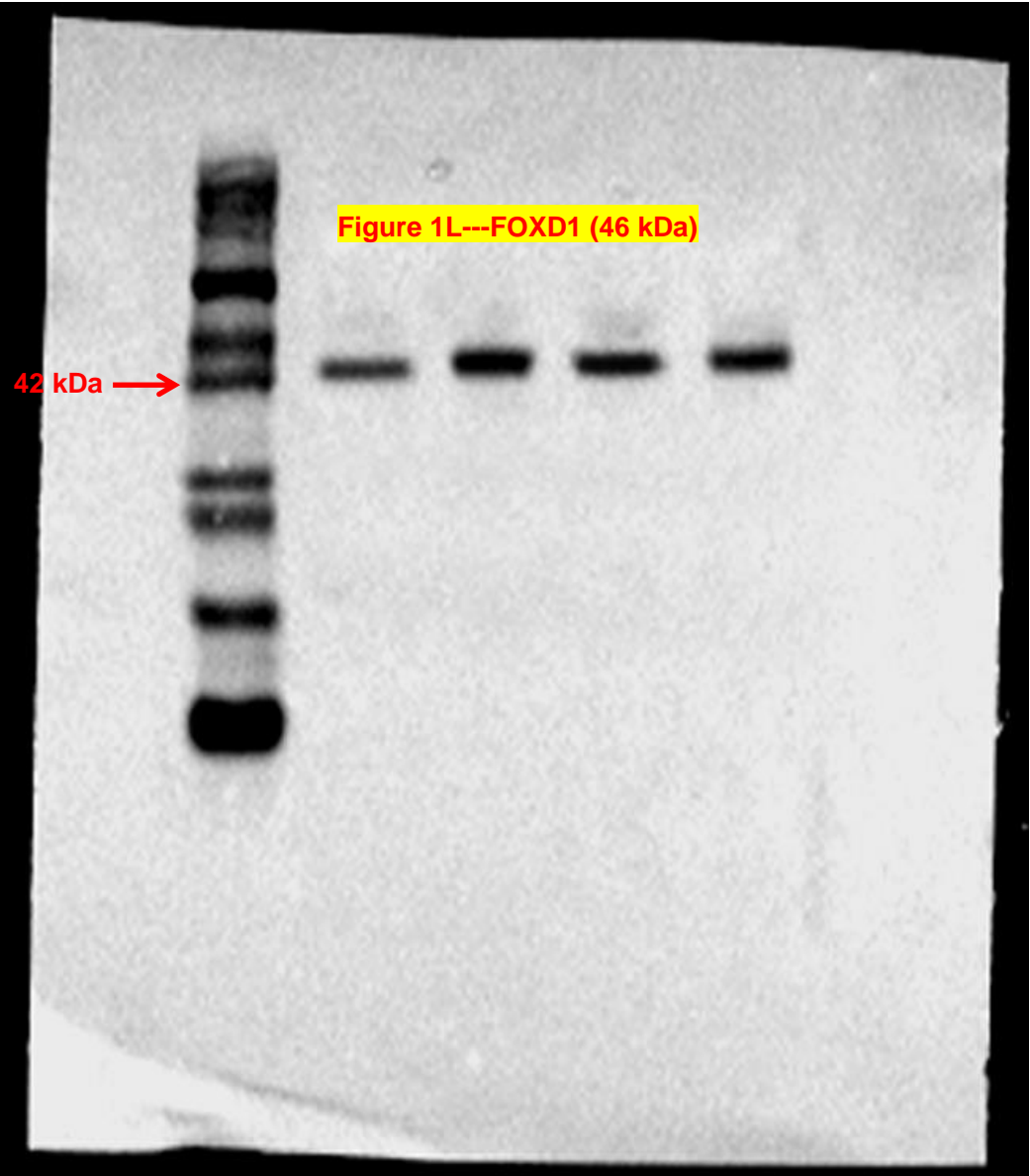

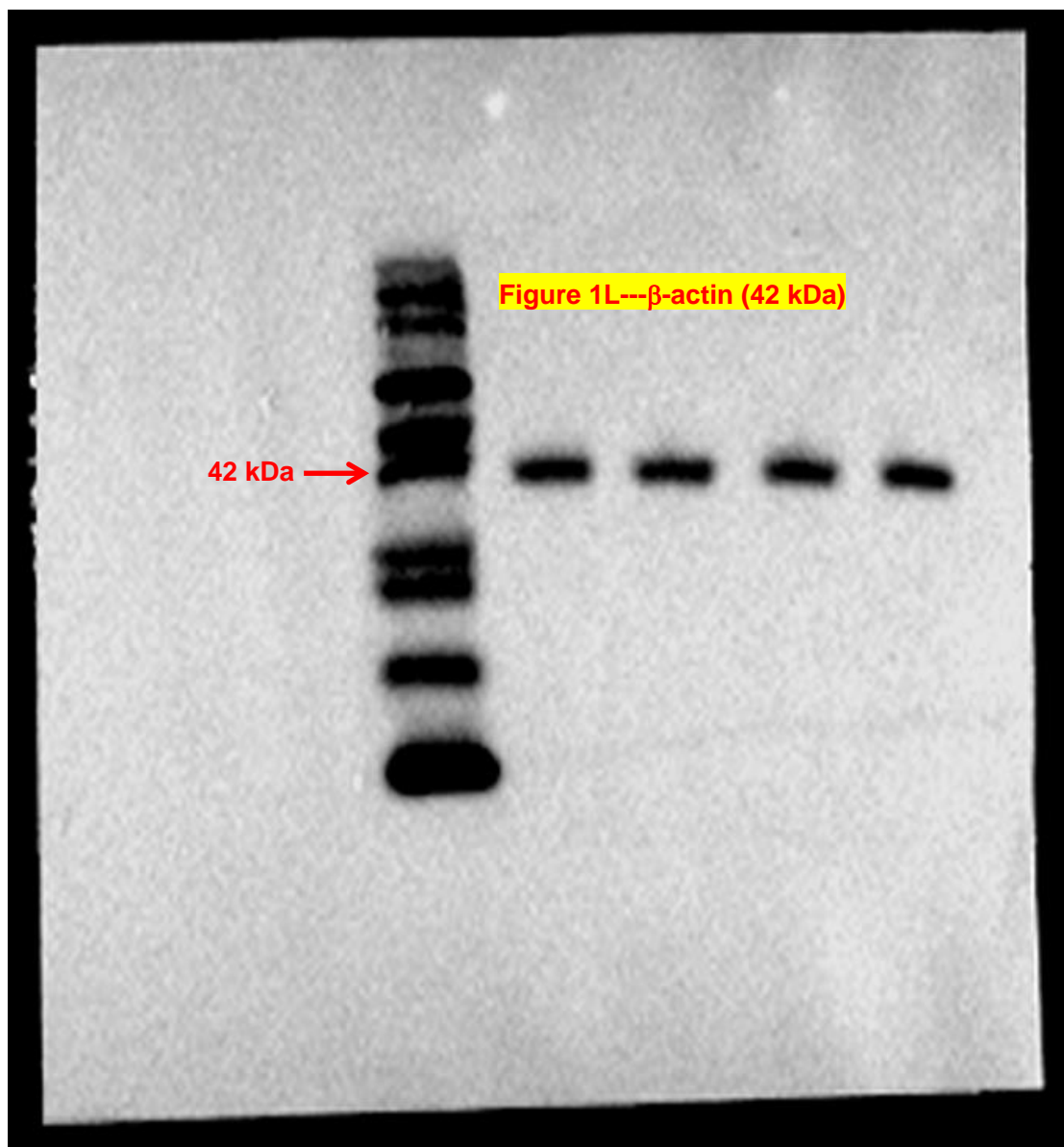

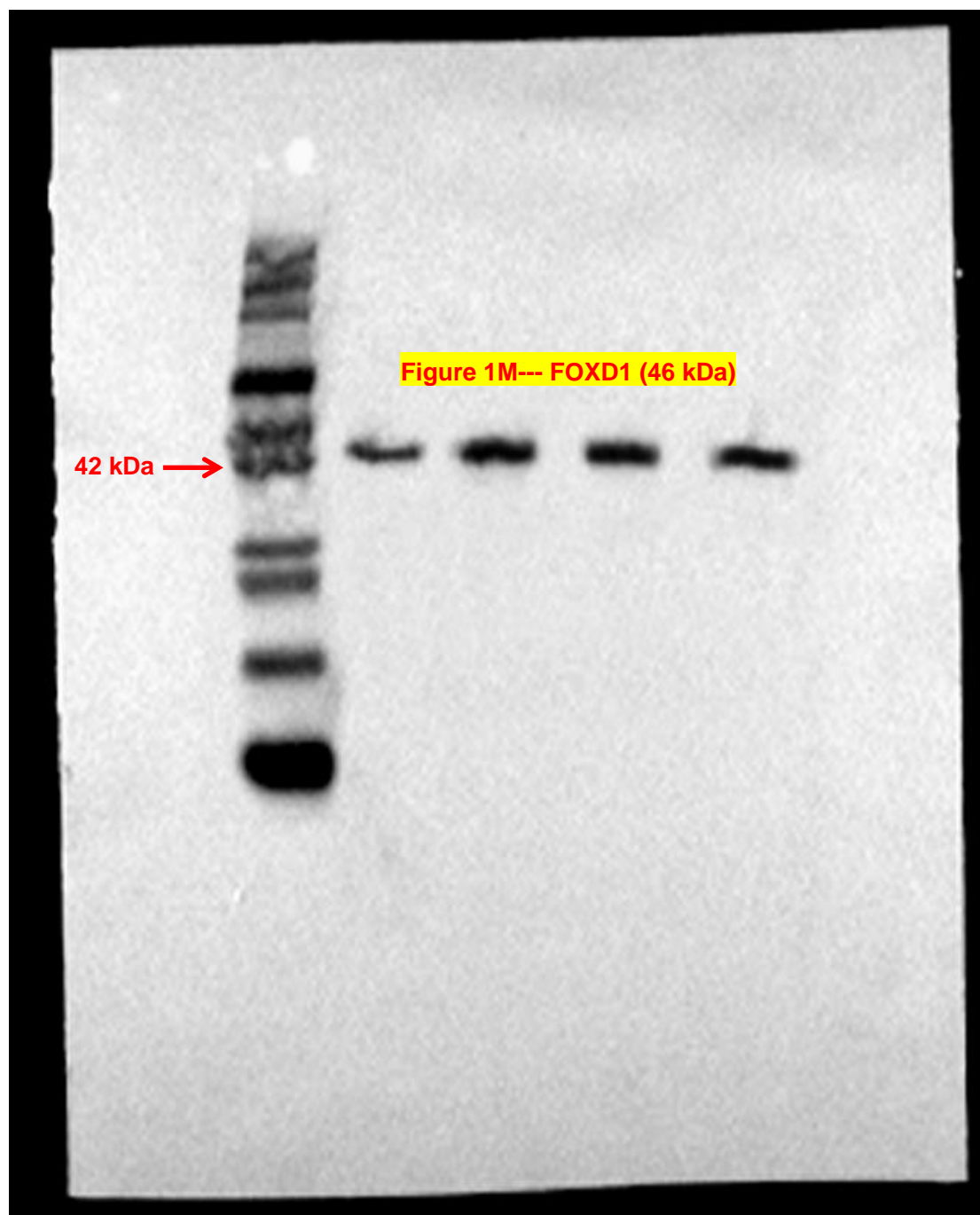

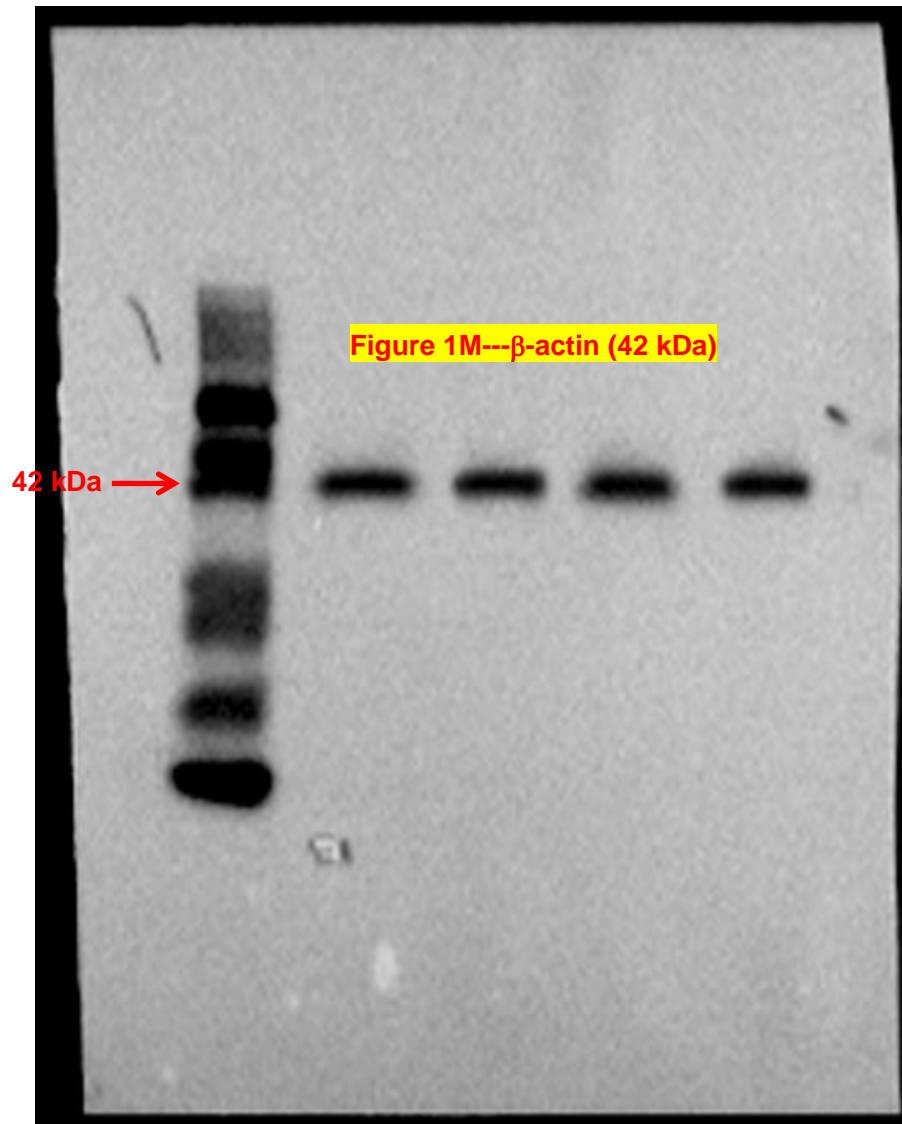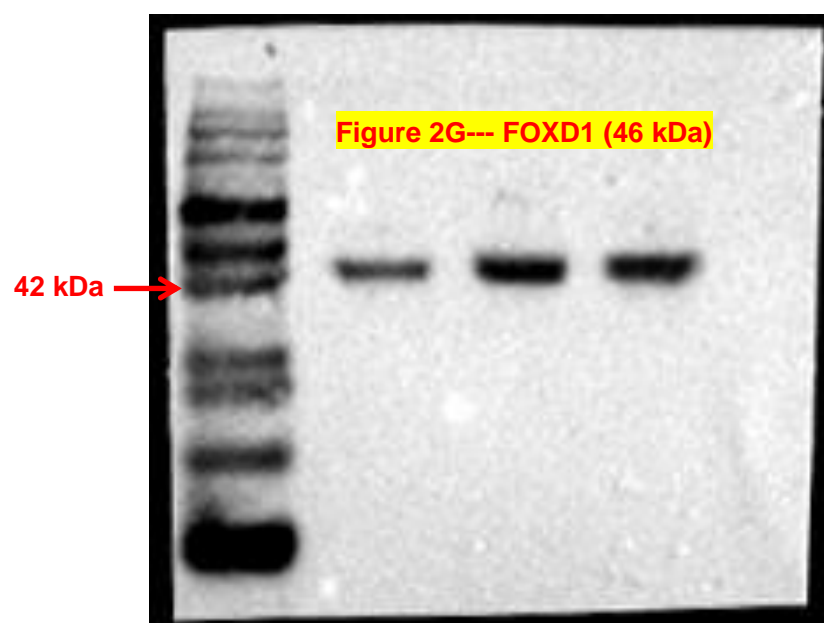

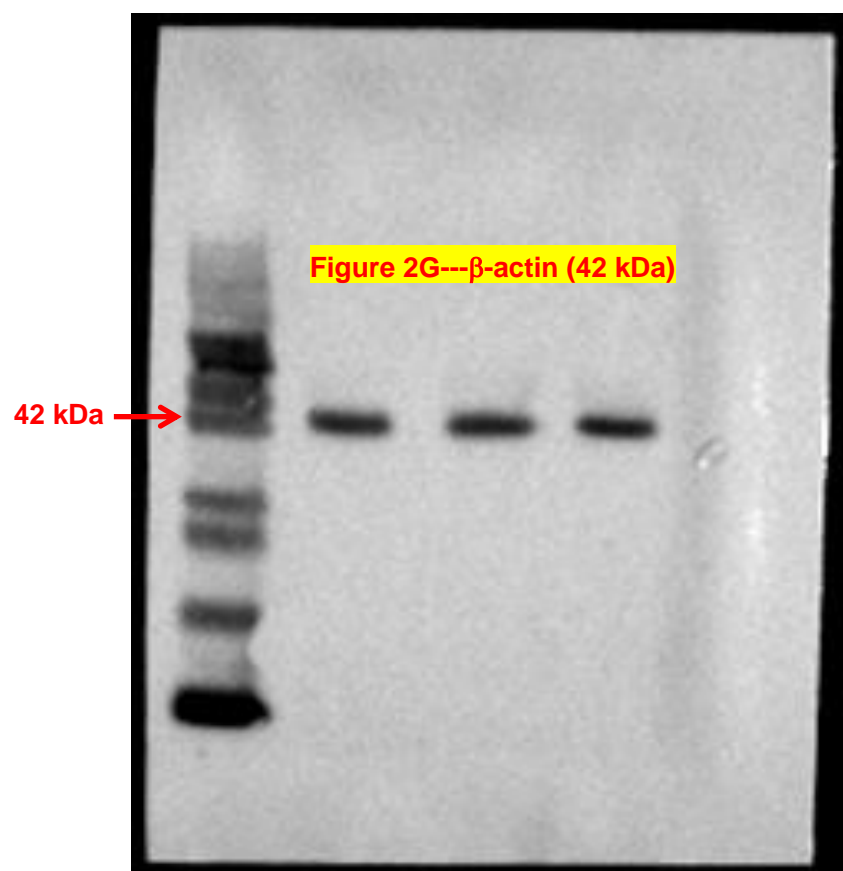

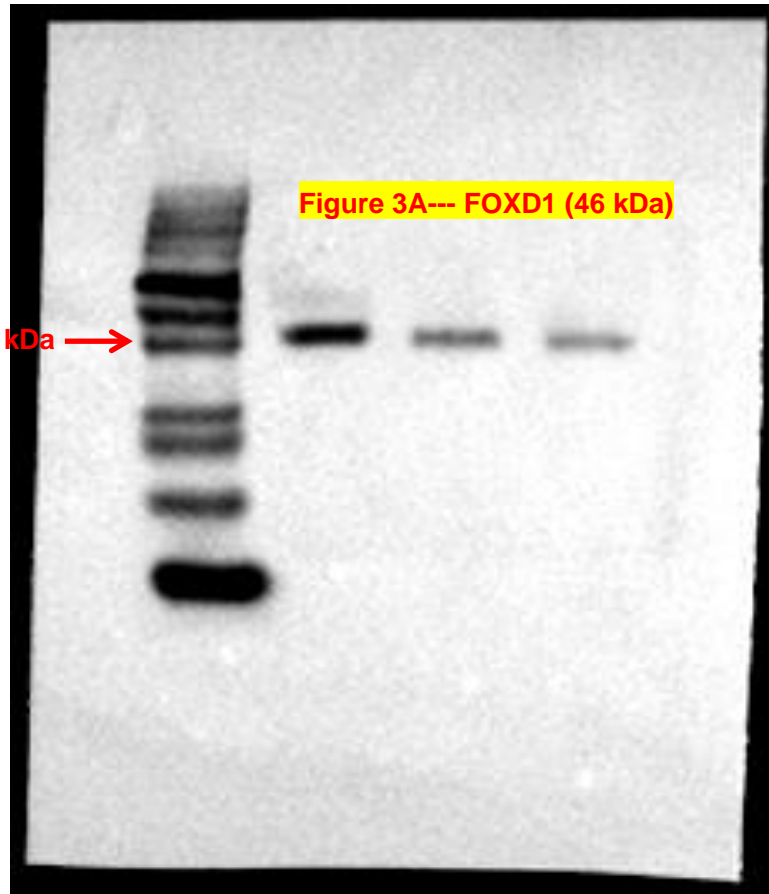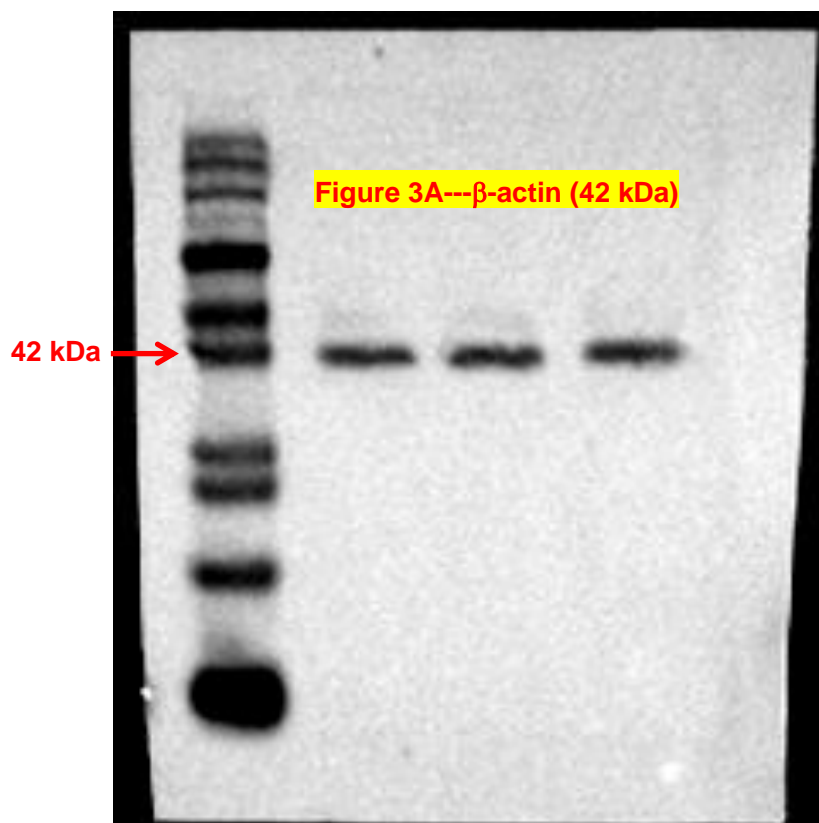

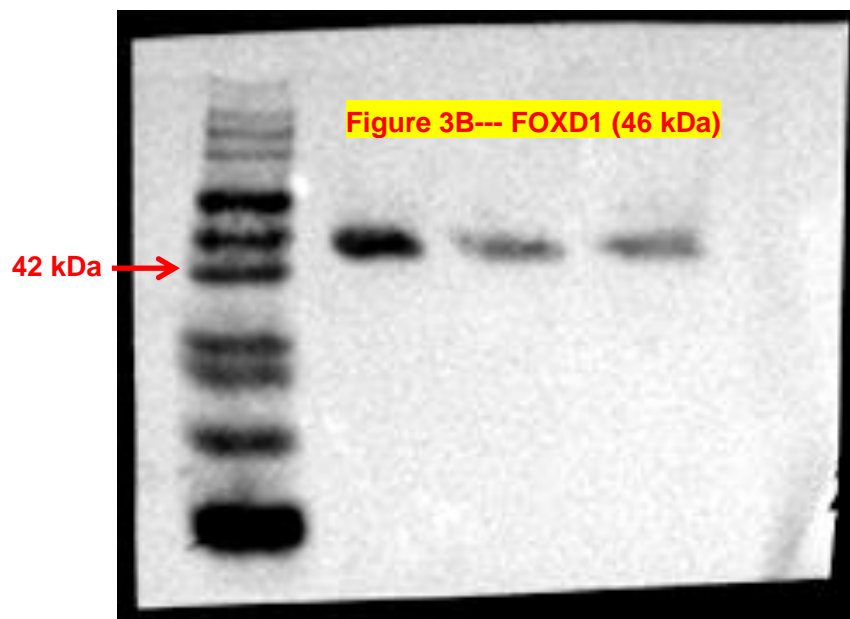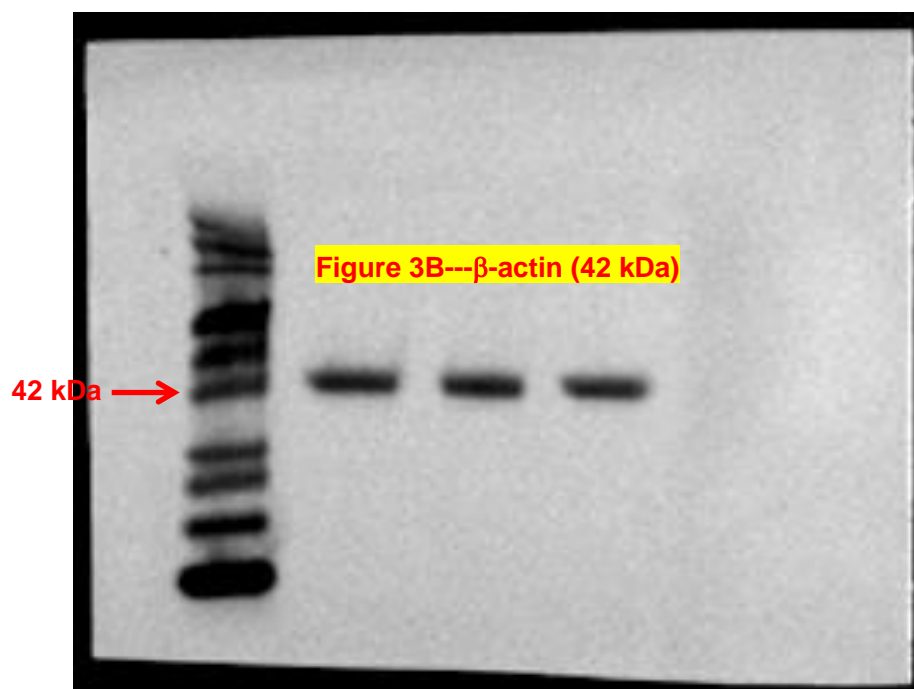

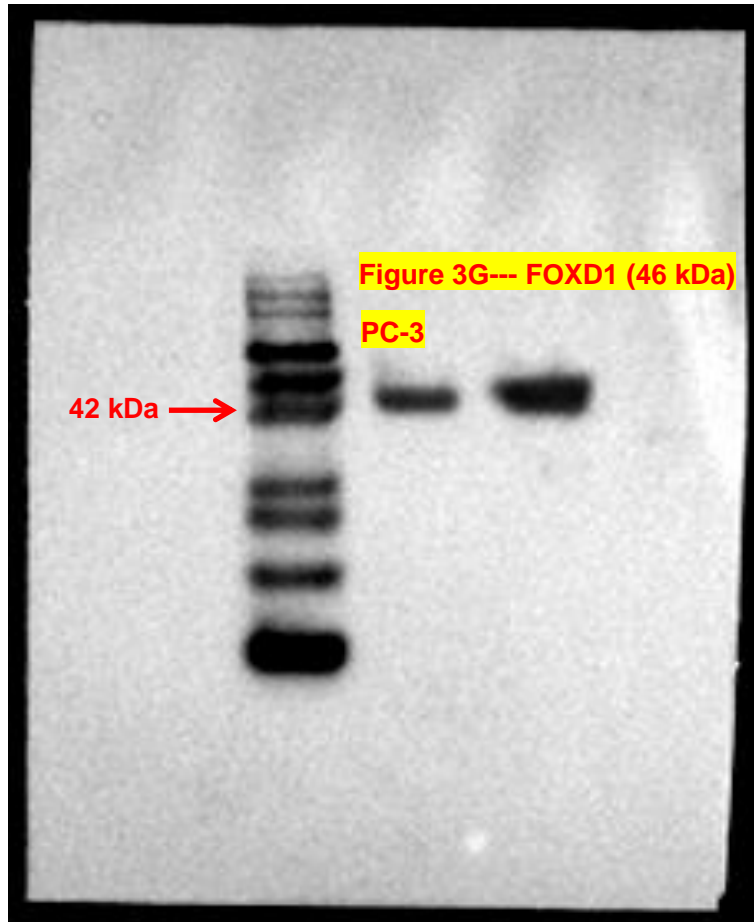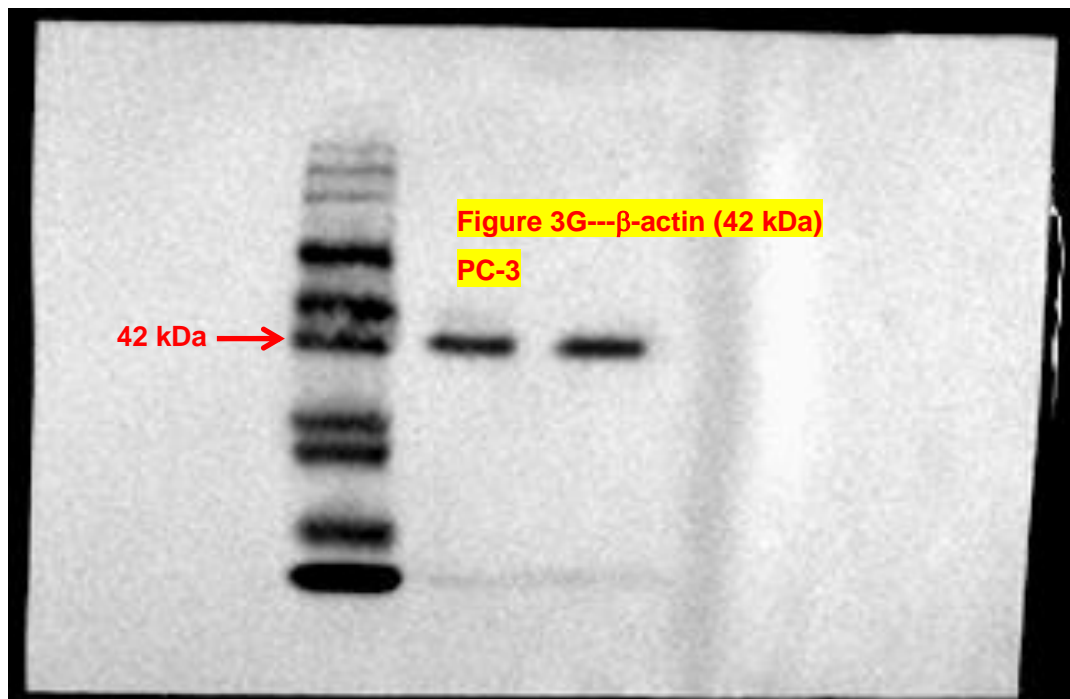

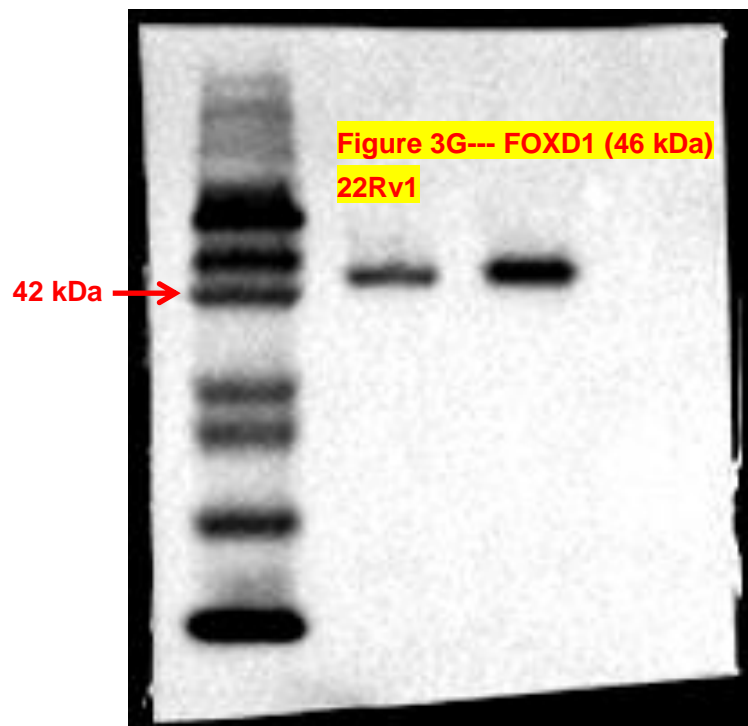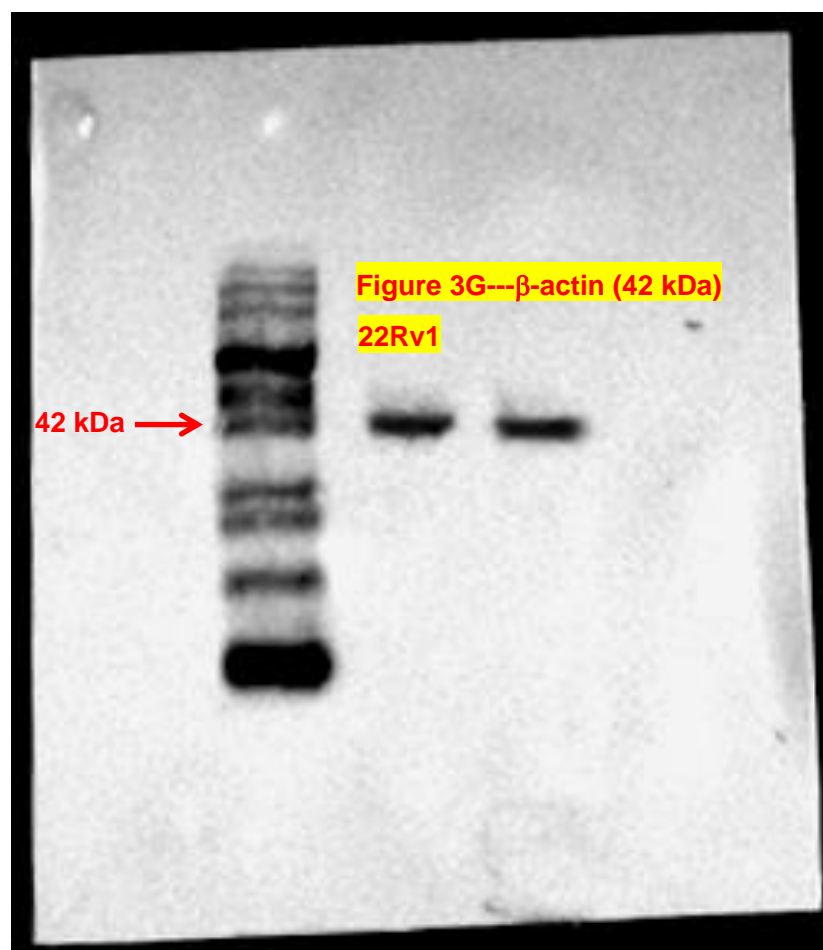

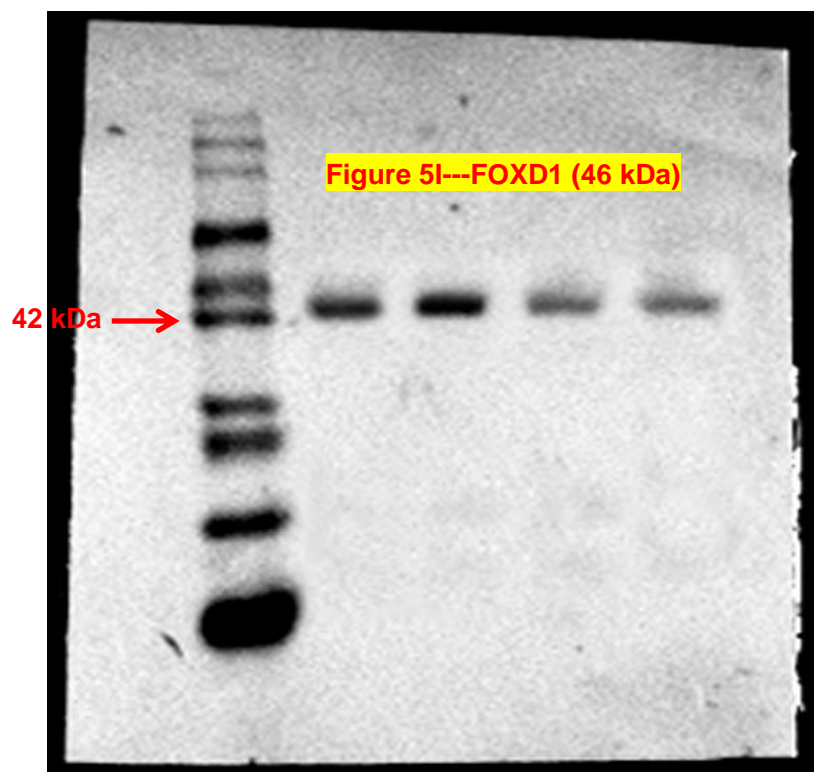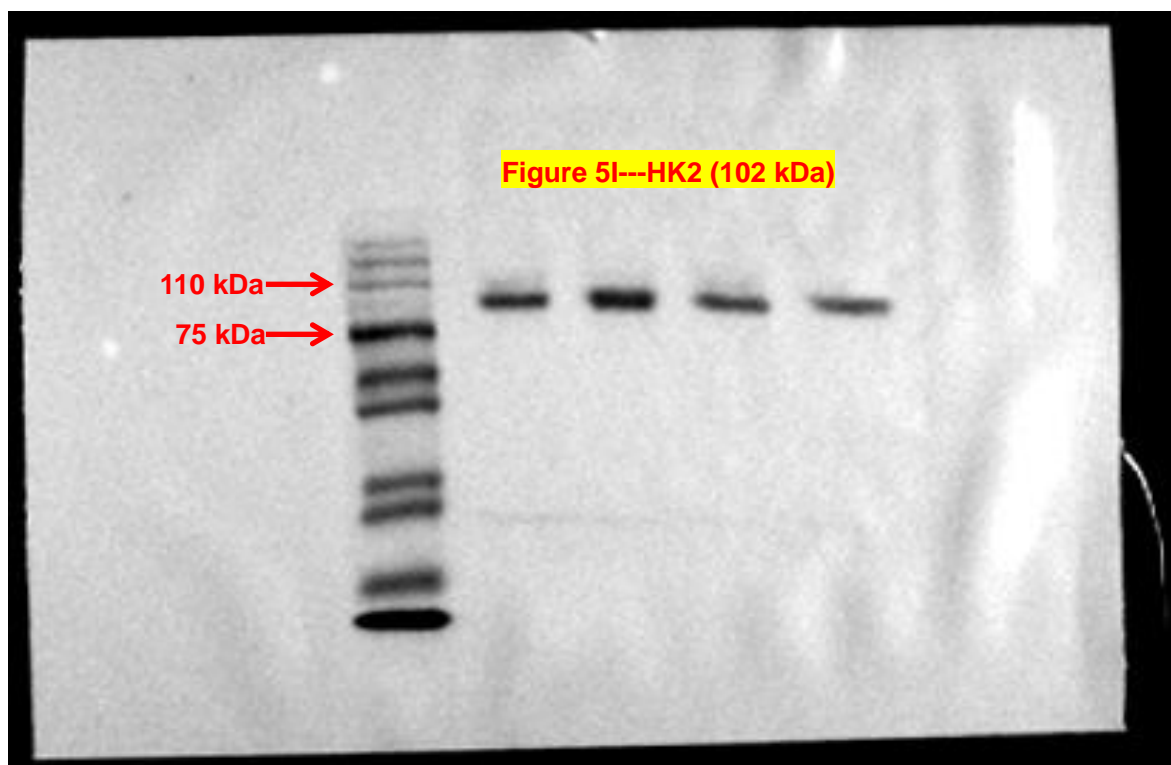

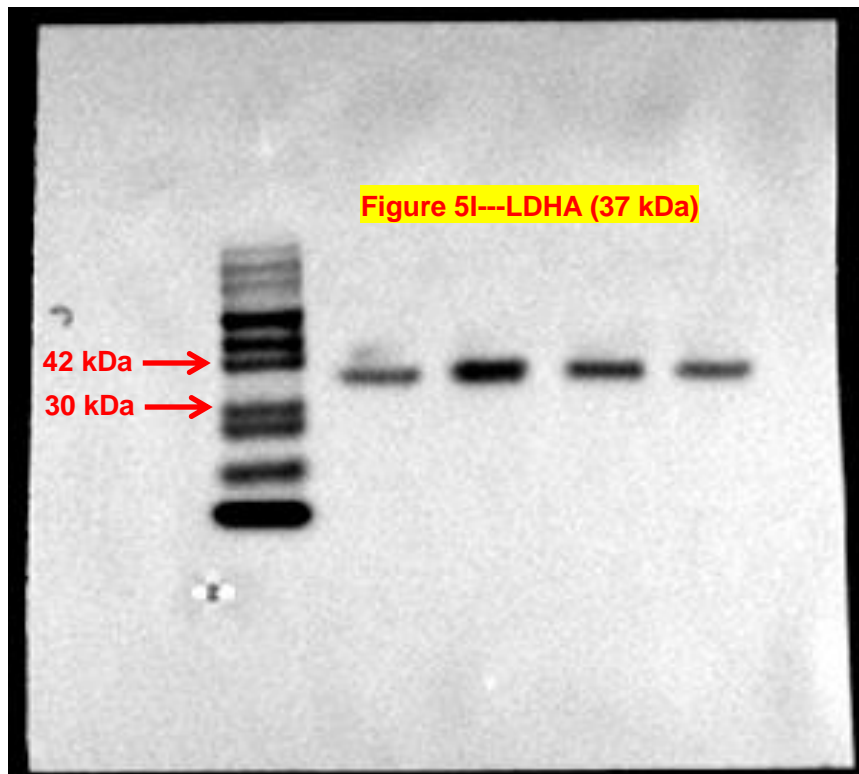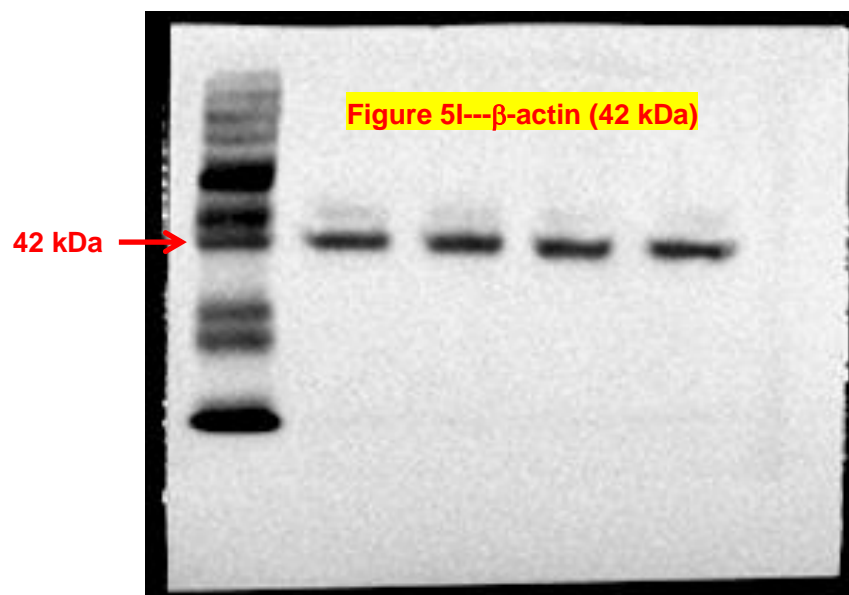

Figure 5J---FOXD1 (46 kDa)

42 kDa →

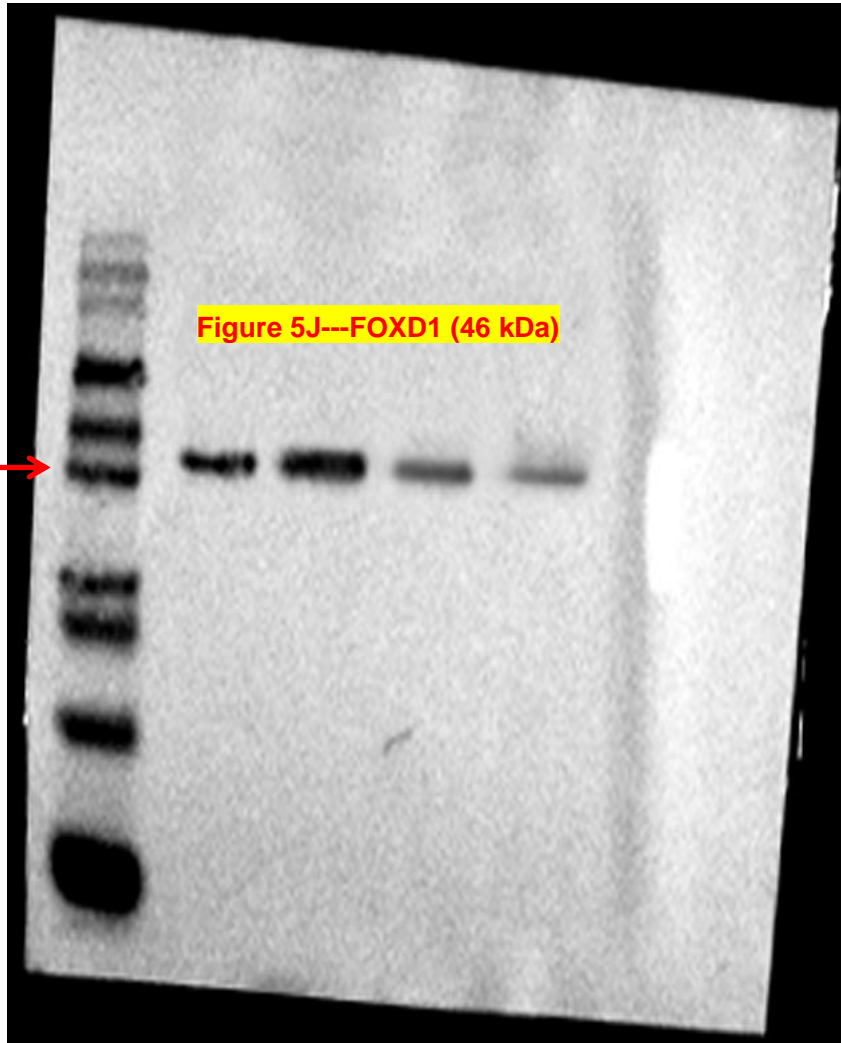

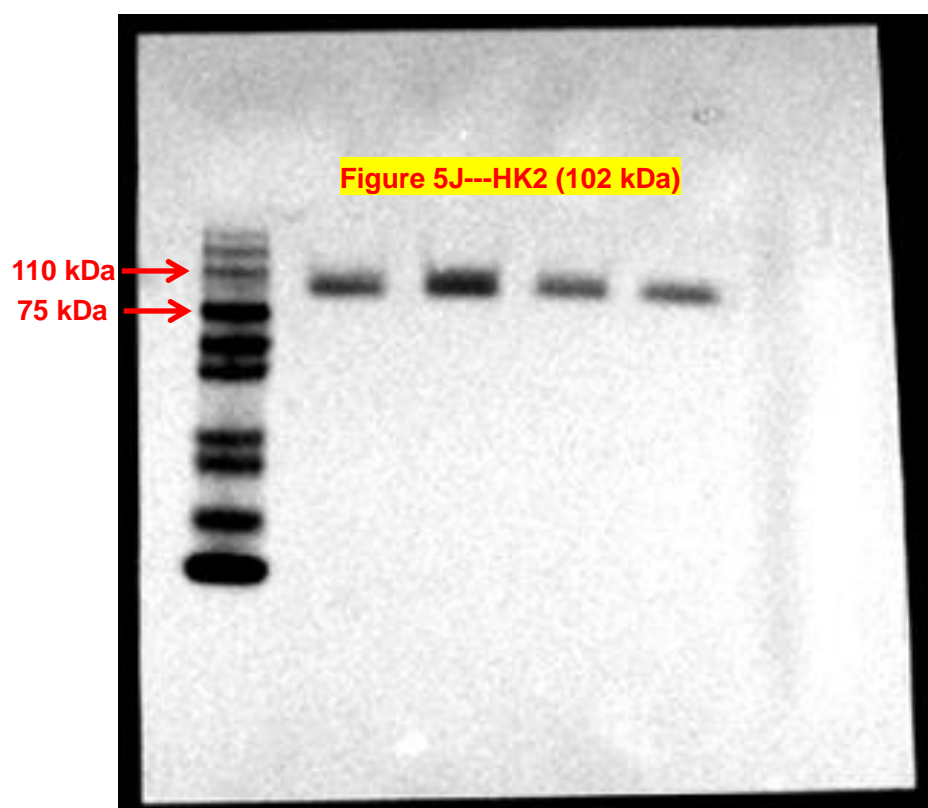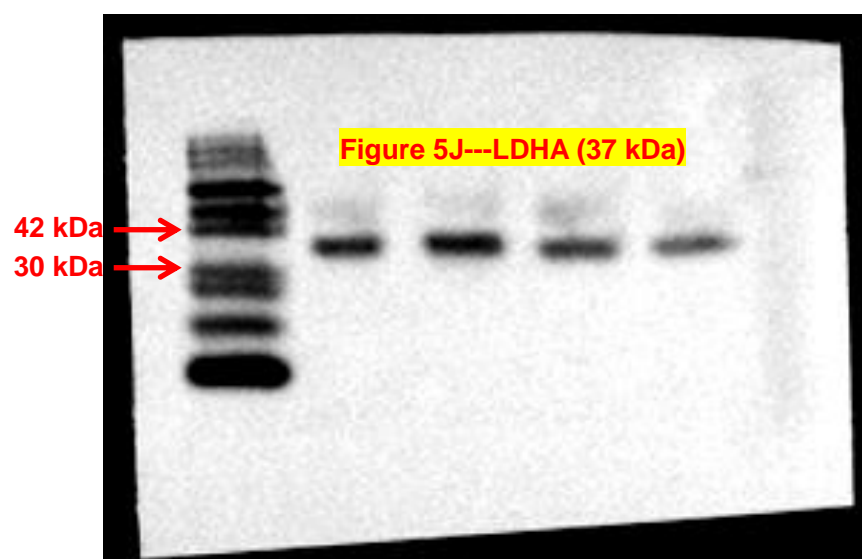

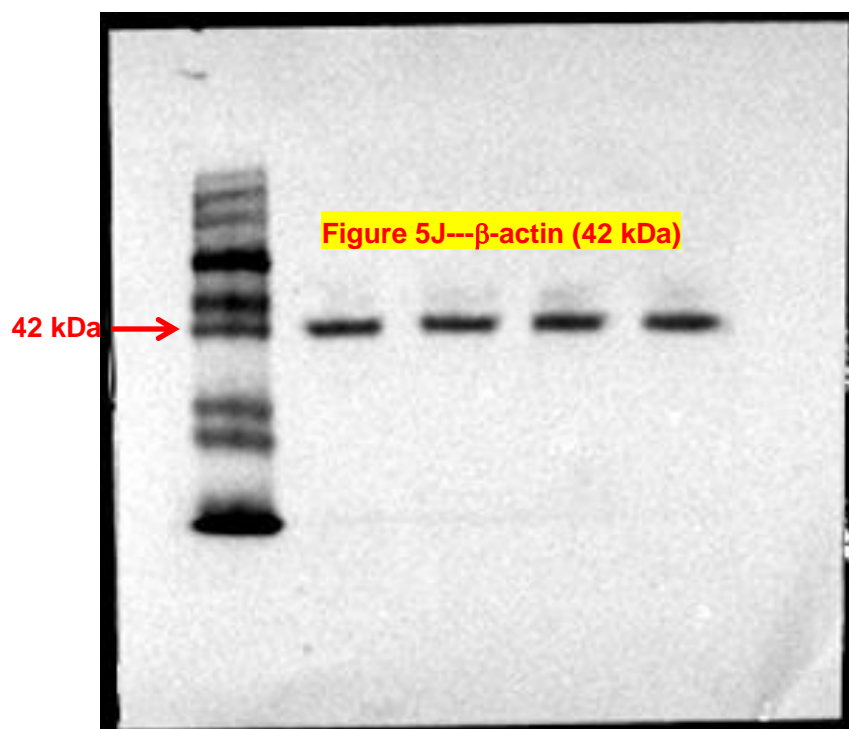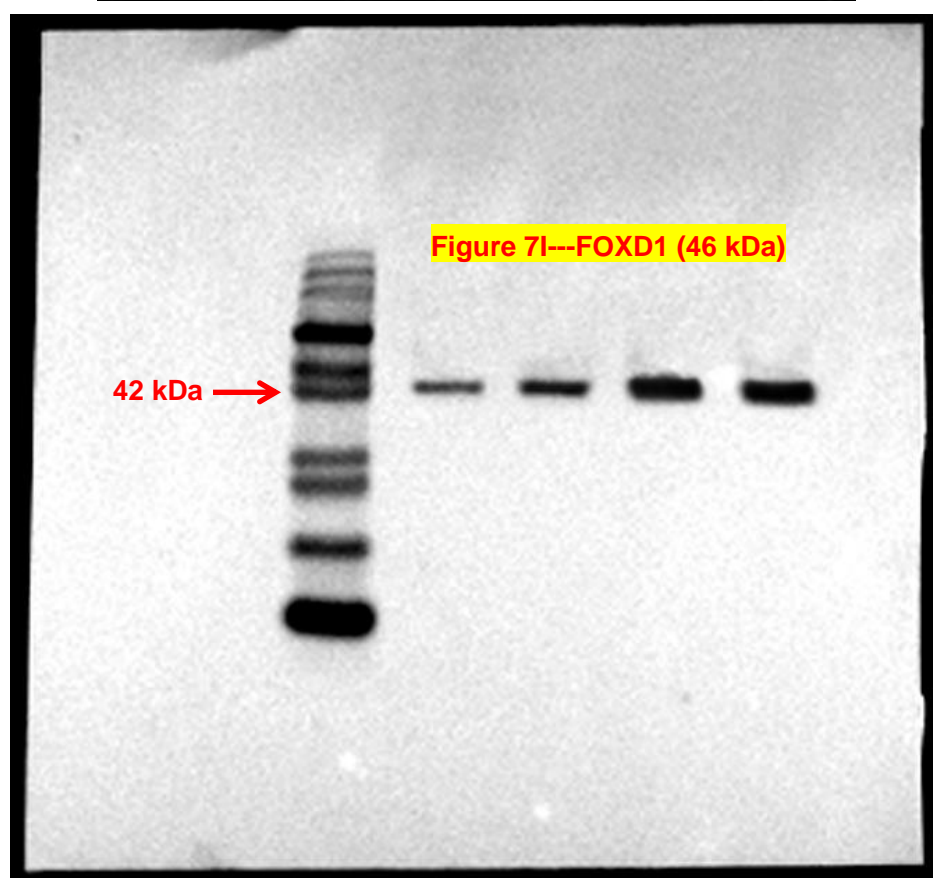

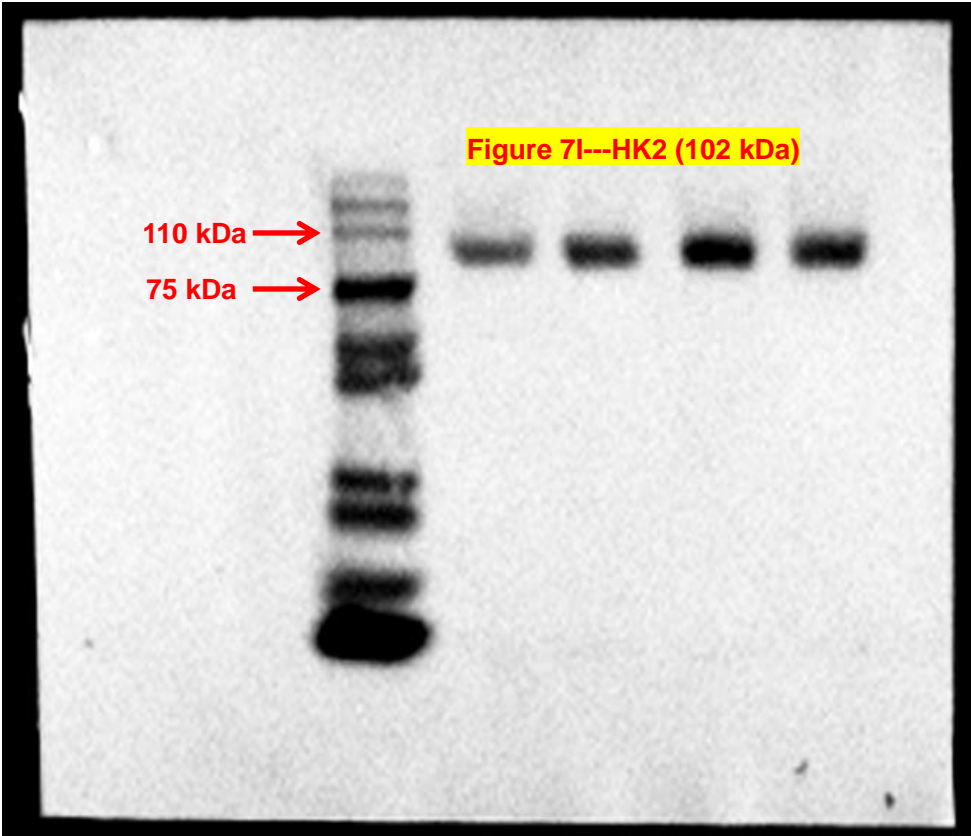

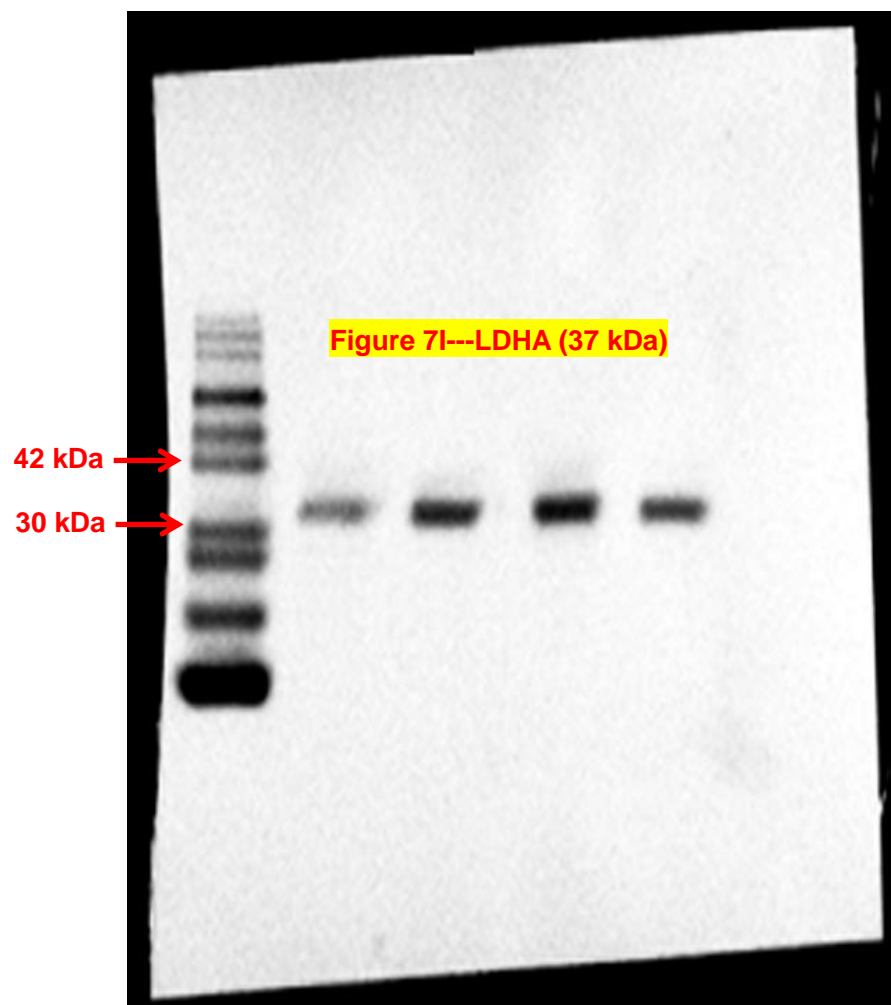

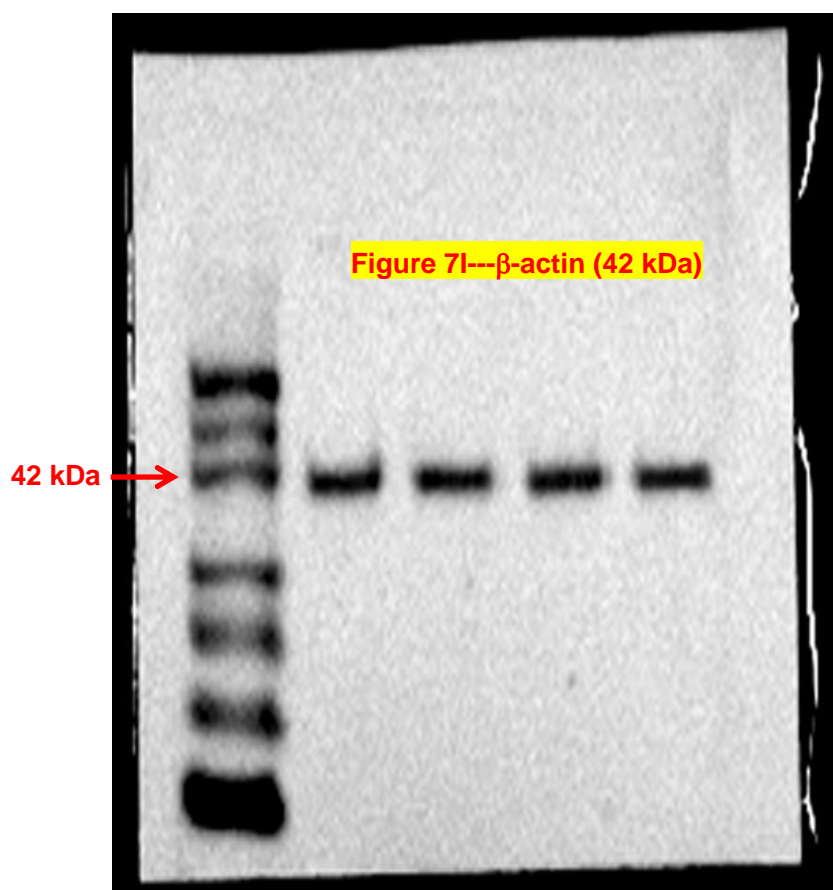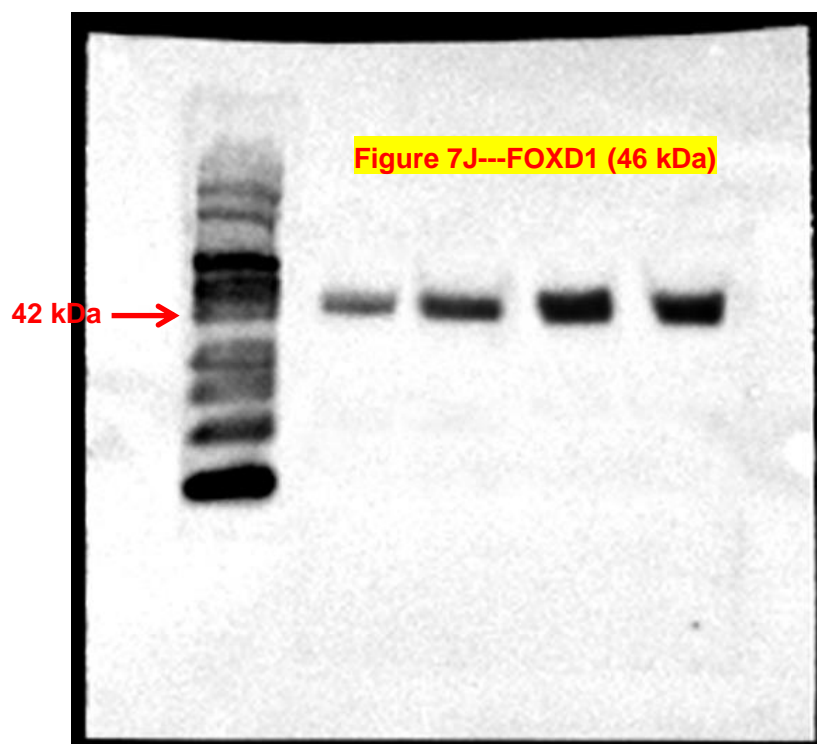

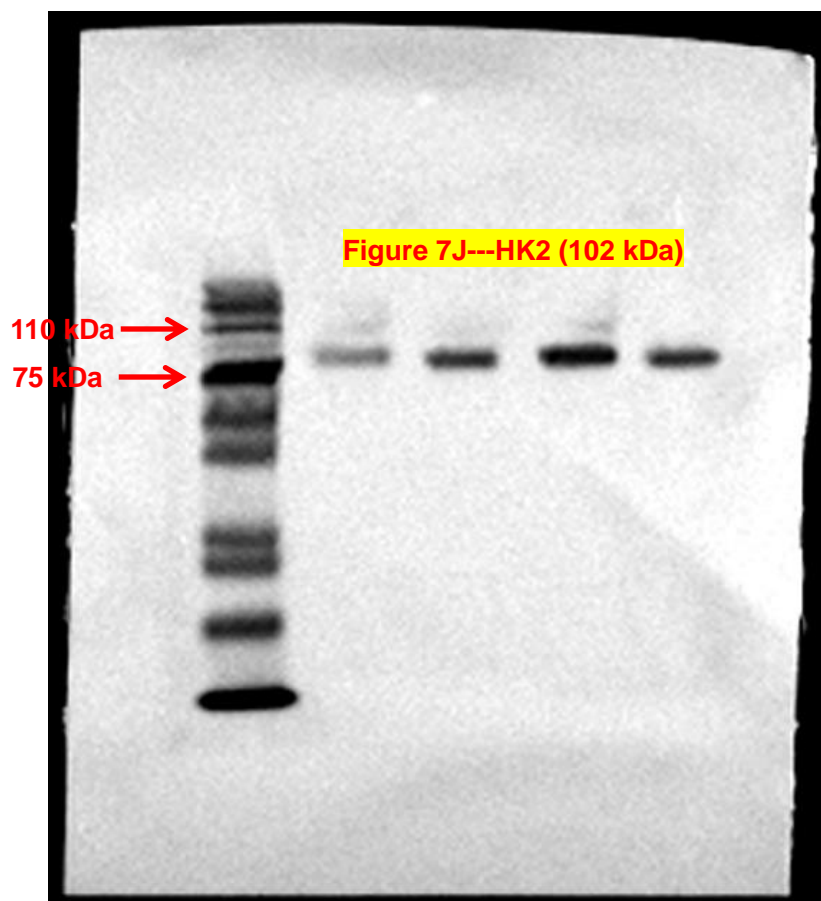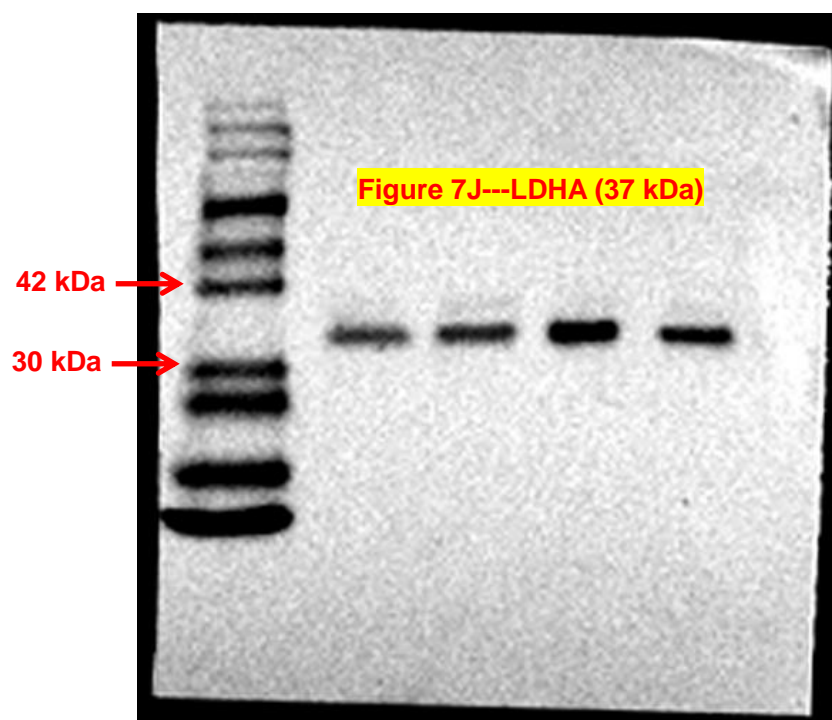

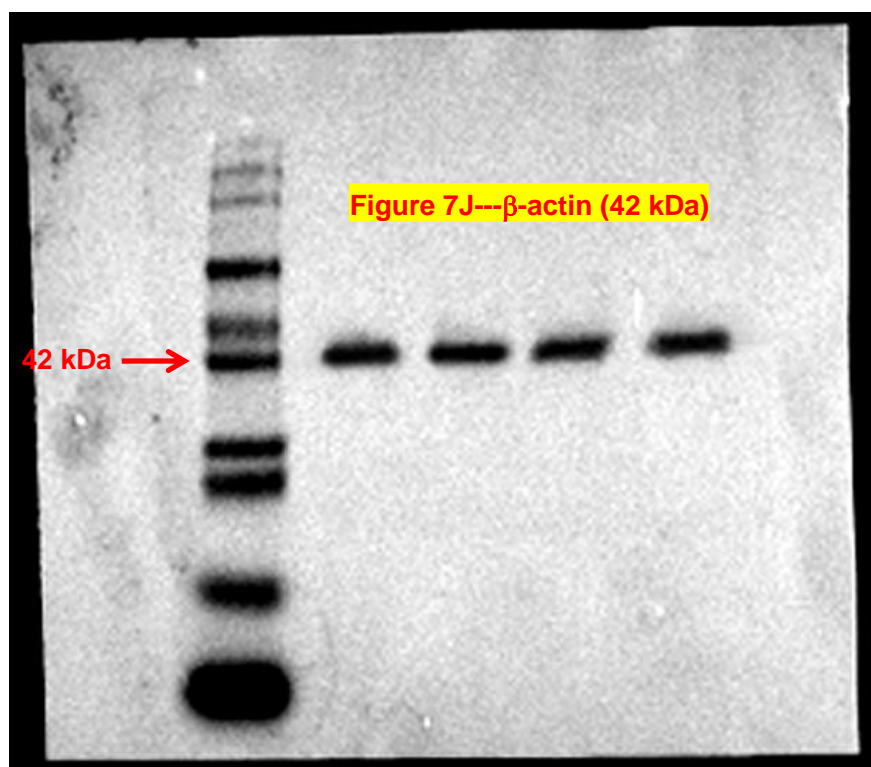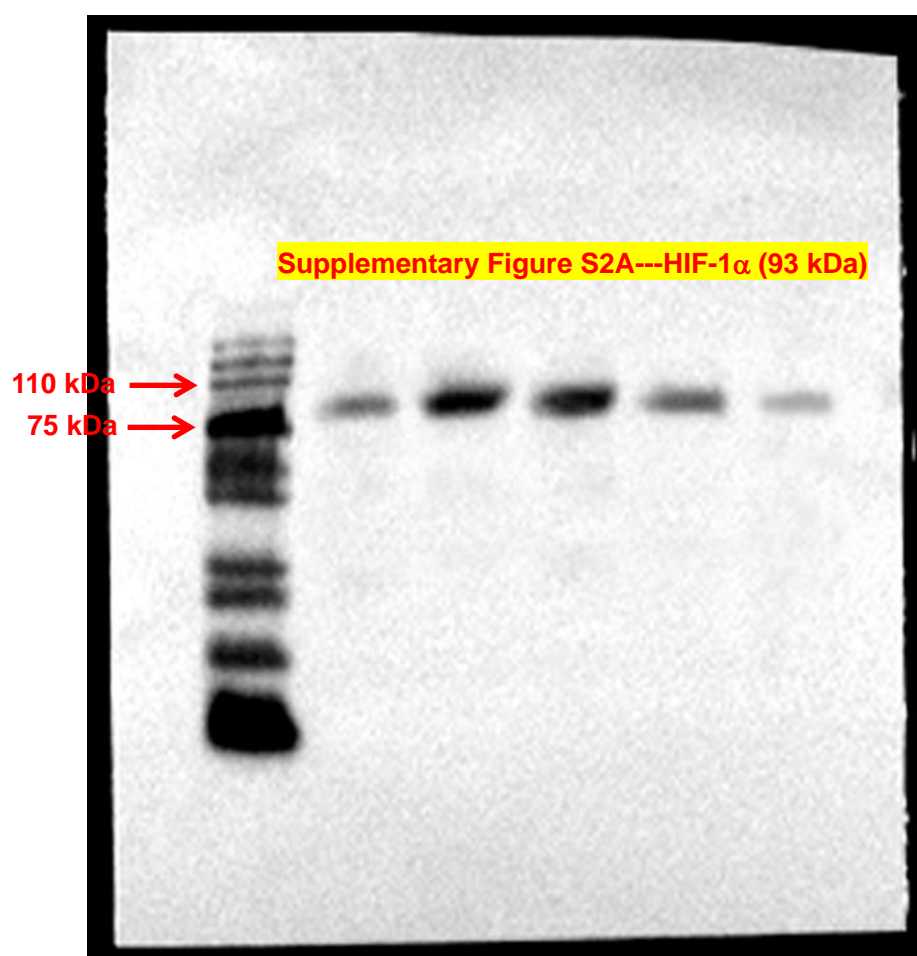

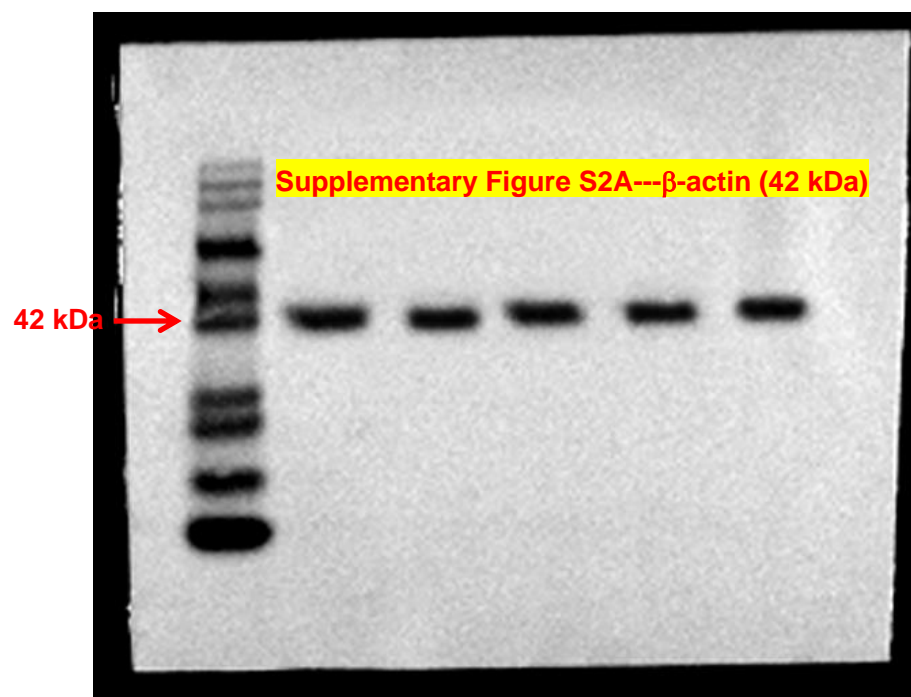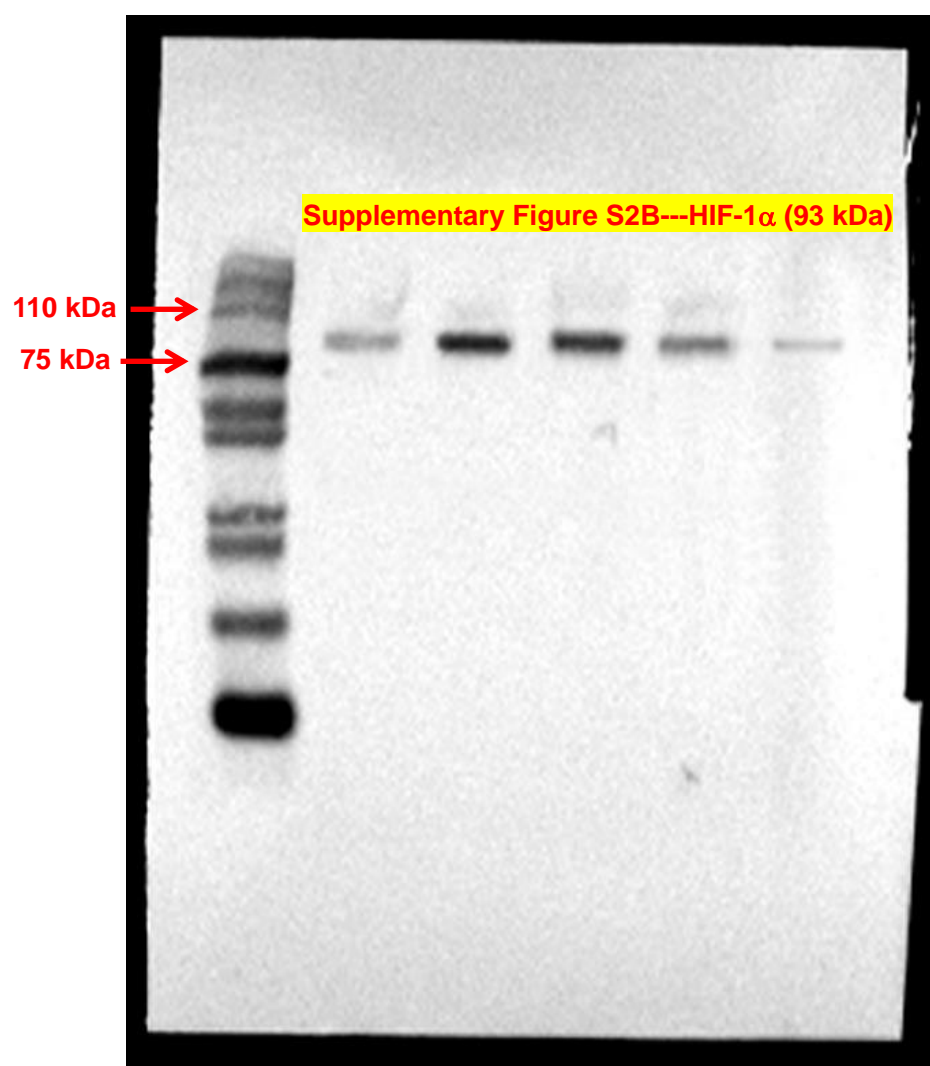

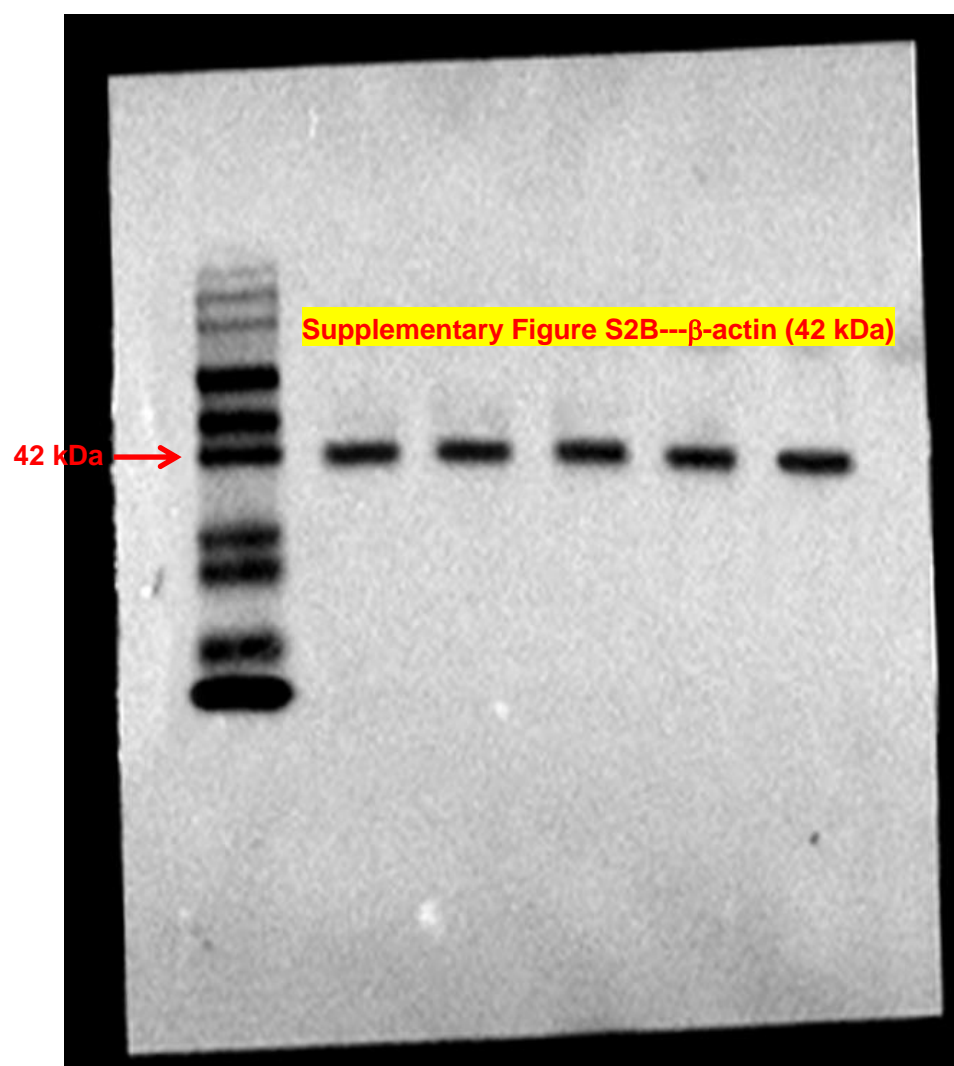

Supplement: Supplementary file 2 — Supplementary Material 2 [file 12896_2025_1061_MOESM2_ESM.pdf]
